# Immune cell topography predicts response to PD-1 blockade in cutaneous T cell lymphoma

**DOI:** 10.1038/s41467-021-26974-6

**Published:** 2021-11-18

**Authors:** Darci Phillips, Magdalena Matusiak, Belén Rivero Gutierrez, Salil S. Bhate, Graham L. Barlow, Sizun Jiang, Janos Demeter, Kimberly S. Smythe, Robert H. Pierce, Steven P. Fling, Nirasha Ramchurren, Martin A. Cheever, Yury Goltsev, Robert B. West, Michael S. Khodadoust, Youn H. Kim, Christian M. Schürch, Garry P. Nolan

**Affiliations:** 1 Department of Microbiology & Immunology, Stanford University School of Medicine, Stanford, CA 94305 USA; 2 Department of Dermatology, Stanford University School of Medicine, Stanford, CA 94305 USA; 3 Department of Pathology, Stanford University School of Medicine, Stanford, CA 94305 USA; 4 Department of Bioengineering, Stanford University Schools of Engineering and Medicine, Stanford, CA 94305 USA; 5 Center for Virology and Vaccine Research, Beth Israel Deaconess Medical Center, Harvard Medical School, Boston, MA 02215 USA; 6 Cancer Immunotherapy Trials Network, Fred Hutchinson Cancer Research Center, Seattle, WA USA; 7 Division of Oncology, Stanford University School of Medicine, Stanford, CA 94305 USA; 8 Department of Pathology and Neuropathology, University Hospital and Comprehensive Cancer Center Tübingen, Tübingen, Germany

**Keywords:** Cancer immunotherapy, T-cell lymphoma, Imaging the immune system, Predictive markers, Cancer microenvironment

## Abstract

Cutaneous T cell lymphomas (CTCL) are rare but aggressive cancers without effective treatments. While a subset of patients derive benefit from PD-1 blockade, there is a critically unmet need for predictive biomarkers of response. Herein, we perform CODEX multiplexed tissue imaging and RNA sequencing on 70 tumor regions from 14 advanced CTCL patients enrolled in a pembrolizumab clinical trial (NCT02243579). We find no differences in the frequencies of immune or tumor cells between responders and non-responders. Instead, we identify topographical differences between effector PD-1^+^ CD4^+^ T cells, tumor cells, and immunosuppressive Tregs, from which we derive a spatial biomarker, termed the *SpatialScore*, that correlates strongly with pembrolizumab response in CTCL. The *SpatialScore* coincides with differences in the functional immune state of the tumor microenvironment, T cell function, and tumor cell-specific chemokine recruitment and is validated using a simplified, clinically accessible tissue imaging platform. Collectively, these results provide a paradigm for investigating the spatial balance of effector and suppressive T cell activity and broadly leveraging this biomarker approach to inform the clinical use of immunotherapies.

## Abstract (web summary)

PD-1 blockade is effective for only a subset of patients with cutaneous T cell lymphomas. Here, the authors report a spatial biomarker that uses immune and cancer cell topography to predict response to PD-1 blockade in this disease.

## Introduction

Cutaneous T cell lymphomas (CTCL) are a heterogeneous group of rare but potentially lethal lymphomas that originate from mature, skin-tropic CD4^+^ T cells^1^. Approximately one-third of CTCL patients present with advanced-stage disease, which has a 5-year survival rate of 28%^2^. With the exception of hematopoietic stem cell transplantation, there are no effective or sustainable therapies for advanced CTCL^3^.

Immune checkpoint inhibitors, such as antibodies against PD-1, restore T cell effector function at the tumor site^4,5^ and promote robust and durable responses in a number of advanced cancers^6–8^. In CTCL, PD-1 and its ligands can be simultaneously expressed on tumor cells, making this pathway an attractive therapeutic target for PD-1 blockade^9–12^.

A recent Cancer Immunotherapy Trials Network (CITN) multicenter phase II clinical trial (NCT02243579) of the anti-PD-1 immunotherapy, pembrolizumab, in advanced cutaneous T cell lymphoma (CTCL) showed that 38% of patients achieved a sustained clinical response, whereas 25% experienced disease progression^13^. Despite these outcome discrepancies, biomarker studies with traditional immunohistochemistry (IHC), gene expression profiling, and mass cytometry did not predict pembrolizumab response^13^. New assays and associated computational tools are critically needed to interrogate the tumor microenvironment (TME) and better predict the clinical response to anti-PD-1 therapies.

Since the immune system acts via coordinated cell-cell associations, it is expected that spatial cellular attributes within the TME prognosticate clinical outcomes. Indeed, recent studies show that immune cells are not randomly distributed within the TME, but rather purposefully organized into cellular neighborhoods and niches that facilitate anti- or pro-tumor functions^14,15^. This raises the question of how PD-1 blockade alters spatial cellular context, and in turn, whether such changes can predict clinical response to pembrolizumab therapy in CTCL. To this end, we interrogate the spatial organization of the CTCL microenvironment in the same cohort of patients enrolled in the previously published pembrolizumab clinical trial, which failed to identify a predictive biomarker of response.

We combine CO-Detection by indEXing (CODEX) multiplexed tissue imaging^15–19^ with transcriptomic analysis using RNA-seq^20,21^ to investigate, in unprecedented detail, 70 tumor regions from 14 advanced CTCL patients sampled before and after pembrolizumab treatment. Characterizing the higher-order tissue structure of the CTCL TME reveal topographical differences in effector PD-1^+^ CD4^+^ T cells, tumor cells, and immunosuppressive Tregs. From this, we derive the *SpatialScore*: a spatial biomarker that correlates strongly with pembrolizumab response and is accurately recapitulated using a clinically accessible multiplexed IHC (mIHC) platform. These results highlight the importance of spatial cellular organization—namely a distancing balance of effector and immunosuppressive T cell activity—for predicting anti-PD-1 immunotherapy response in CTCL. Additionally, this biomarker discovery approach can be readily applied to guide the clinical use of cancer immunotherapies in CTCL and a range of other tumor types.

## Results

### Patient cohort and multimodal experimental approach

To deeply characterize and interrogate the CTCL TME in its native context, we analyzed pre- and post-treatment biopsies from 14 heavily pre-treated patients with advanced-stage CTCL (i.e., mycosis fungoides and Sézary syndrome) enrolled in the CITN-10 clinical trial cohort (NCT02243579), who received pembrolizumab every 3 weeks for up to 2 years (Supplementary Fig. 1a–b, Supplementary Table 1a)^13^. The post-treatment biopsies were collected at several timepoints, as detailed per patient in Supplementary Fig. 1c and Supplementary Table 1a–b. For this study, 10 patients from the original 24 patient cohort were excluded due to insufficient formalin-fixed paraffin-embedded (FFPE) sample material.

**Fig. 1:**
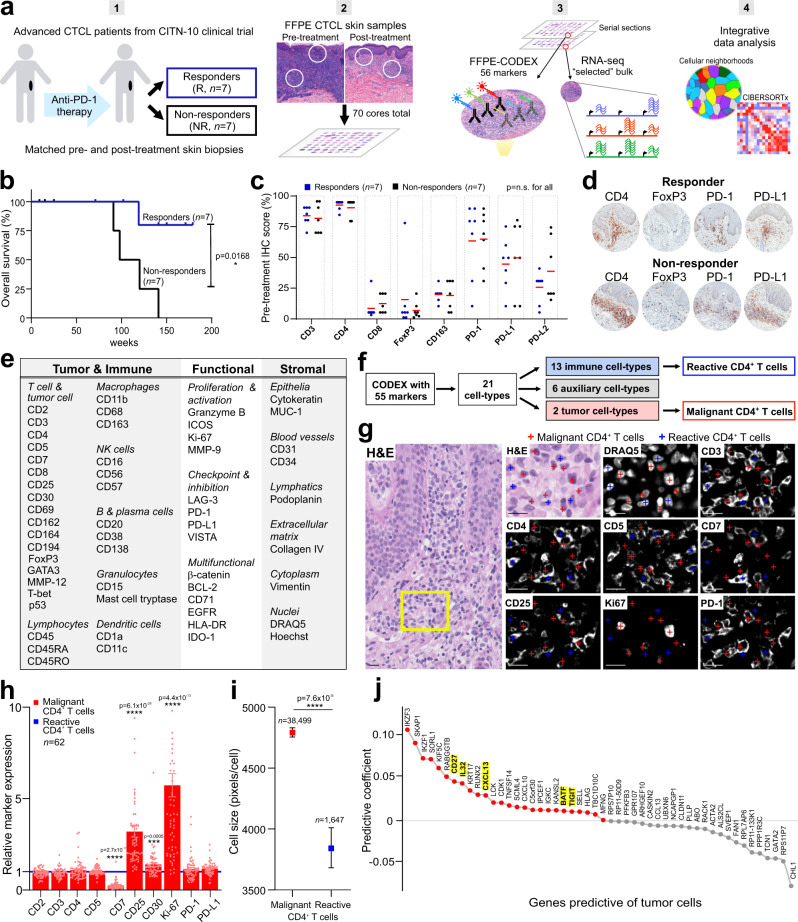
Discrimination of malignant and reactive CD4^+^ T cells in the CTCL TME. **a** Workflow for sample preparation, CODEX, RNAseq, and computational analyses. **b** Kaplan-Meier overall survival curve, comparing responders and nonresponders (hazard ratio 0.0969 responder/nonresponder; *p* value calculated by log-rank test). **c** Pretreatment IHC protein marker expression per patient in responders and nonresponders (mean, red bar). *P* values calculated by two-sided Wilcoxon’s rank-sum tests (*p* = not significant (n.s.) for all comparisons). **d** Representative pretreatment IHC images for select markers from a responder (top) and nonresponder (bottom). **e** CODEX antibody panel (see also Supplementary Fig. 1e). **f** Identification of 21 cell types by clustering (see also Supplementary Fig. 2a, b). **g** Visual verification of reactive (blue crosses) versus malignant (red crosses) CD4^+^ T cells in CTCL tissue. Scale bars, 20 µm. **h**, Mean expression of select markers on all malignant (red bars, mean ± s.e.m.) relative to reactive (blue line) CD4^+^ T cells per tissue microarray spot (pink circles); cores were excluded if they contained <5 CD4^+^ T cells. *P* values calculated by two-sided Wilcoxon’s rank-sum tests. **i** Cell size, measured in pixels/cell, of all malignant (red square) and reactive (blue square) CD4^+^ T cells (mean ± s.e.m.). *P* value calculated by a two-sided Wilcoxon’s rank-sum test. **j** Ranking genes most predictive of tumor cells per tissue microarray spot using an L1-regularized linear model. Red-colored genes have positive predictive coefficients (i.e., most likely to represent tumor cells); gray-colored genes have negative predictive coefficients (i.e., less likely to represent tumor cells). Known CTCL marker genes are highlighted in yellow. Source data are provided as a Source Data file.

An FFPE tissue microarray was created from the 14 patient samples (Fig. 1a.1), which comprised 70 patient-matched pre- and post-treatment skin tumors (Fig. 1a.2). The tissue microarray spots were selected from the most infiltrated regions of the skin biopsies to preclude sampling areas that were void of tumor and immune cell types. The reproducibility of the CODEX approach, as shown for cell densities (# of cells/mm^2^) per tissue microarray spot, is demonstrated across three experiments (Supplementary Fig. 1d). CODEX multiplexed protein imaging identified 117,170 cells in the tissue microarray (Fig. 1a.3, Supplementary Data 1). These results were integrated with 64 tissue transcriptomes obtained from serial sections using laser capture microdissection and Smart-3Seq^20^ (i.e., RNA-seq) (Fig. 1a.3, Supplementary Data 2, Supplementary Data 3). Integrative computational analyses, including cellular neighborhood assessment^15^ and CIBERSORTx (CSx)^21^, were then used to profile the molecular dynamics of the CTCL microenvironment and identify a predictive biomarker of anti-PD-1 immunotherapy (Fig. 1a.4). Comparing the biomarker assays applied to this cohort (Supplementary Fig. 1a), highlights the ability of multiplexed spatial assays to extract new features and predict therapeutic response.

Therapeutic response to pembrolizumab was assessed by consensus global response criteria^22^. No significant differences were observed at baseline between responders and nonresponders for patient demographics, cancer type/stage, or treatment history (Supplementary Fig. 1b). Clinical outcomes were significantly different between patient groups. Responders had a significant improvement in their overall skin response compared to nonresponders, as measured by the modified Severity Weighted Assessment Tool (mSWAT)^23^ (Supplementary Fig. 1b). Overall survival was significantly longer in responders than nonresponders (i.e., nonresponders had a median survival of 109 weeks after treatment initiation, whereas all but one responder was alive at the median follow-up time of 142 weeks) (Fig. 1b). The expression of key T cell, macrophage and PD-1 signaling markers was assessed by standard single-plex IHC for each patient at baseline (Fig. 1c). No differences were observed for these eight markers (Fig. 1c), as shown for CD4, FoxP3, PD-1, and PD-L1 (Fig. 1d). These results demonstrate that the patient characteristics for the current study (*n* = 14 patients) do not differ from the full clinical trial cohort (*n* = 24 patients)^13^.

### Discrimination of malignant and reactive CD4^+^ T cells in the CTCL microenvironment

Detecting a biomarker based on the spatial context of tumor and immune cells requires that malignant and reactive CD4^+^ T cells can be distinguished. This is a major challenge in CTCL, because the cell type of origin (e.g., effector memory T cells (T_EM_), central memory T cells (T_CM_), tissue-resident memory T cells (T_RM_))^24^, expression of T cell markers (e.g., CD2, CD3, CD4, CD5, CD7, CD25, FoxP3), and clonality^25^ can be shared by both cell types. Furthermore, no protein marker has yet been identified as 100% specific for CTCL tumor cells. Using a 56-marker CODEX panel (Fig. 1e, Supplementary Fig. 1e, Supplementary Table 2), unsupervised machine learning, and manual curation based on marker expression, tissue localization, and morphology, 21 unique cell types were identified and validated (Fig. 1f, Supplementary Fig. 2a, b), including reactive CD4^+^ T cells and malignant CD4^+^ T cells (i.e., tumor cells). Comparing the fluorescent staining of reactive CD4^+^ T cells (blue crosses) and malignant CD4^+^ T cells (red crosses) showed that in tumor cells CD7 was decreased and CD25 and Ki-67 were increased (Fig. 1g), consistent with an advanced CTCL phenotype^26^. Quantifying these expression differences for tumor cells relative to reactive CD4^+^ T cells revealed fold-changes of 0.26 for CD7, 1.40 for CD30, 3.20 for CD25, and 5.73 for Ki-67 (Fig. 1h). Tumor cells were also larger than reactive CD4^+^ T cells (Fig. 1i), in line with the previous reports^27^. While one patient’s tumor cells had large cell transformation, the average size of those tumor cells were not significantly different from those of the other 13 patients. Additionally, we identified genes predictive of the frequency of tumor cells per tissue microarray spot by fitting an L1-regularized linear model to bulk RNA-seq data. This confirmed that higher expression of several known CTCL marker genes, including *CD27*, *IL-32*, *CXCL13*, *BATF*, and *TIGIT*^28–32^, was associated with spots with higher frequencies of tumor cells (Fig. 1j, see yellow highlighted genes). Notably, CTCL tumor cells can also express FoxP3^33^, as seen for one patient in this cohort. Our clustering approach identified this population of malignant FoxP3^+^ CD4^+^ T cells, which differed significantly from Tregs, with lower CD4, CD7, CD25, and FoxP3 marker expression and larger cell size (Supplementary Fig. 2d). Given the heterogeneity of CTCL, the marker expression profile of tumor cells may differ between cohorts, especially if the patients were heavily pre-treated. However, our findings show that multiplexed imaging can discriminate tumor cells from reactive CD4^+^ T cells at the single-cell level, which is supported by protein expression differences, cell size measurements, and gene expression profiling.

### Deep profiling of the CTCL TME in response to anti-PD-1 immunotherapy

**Fig. 2:**
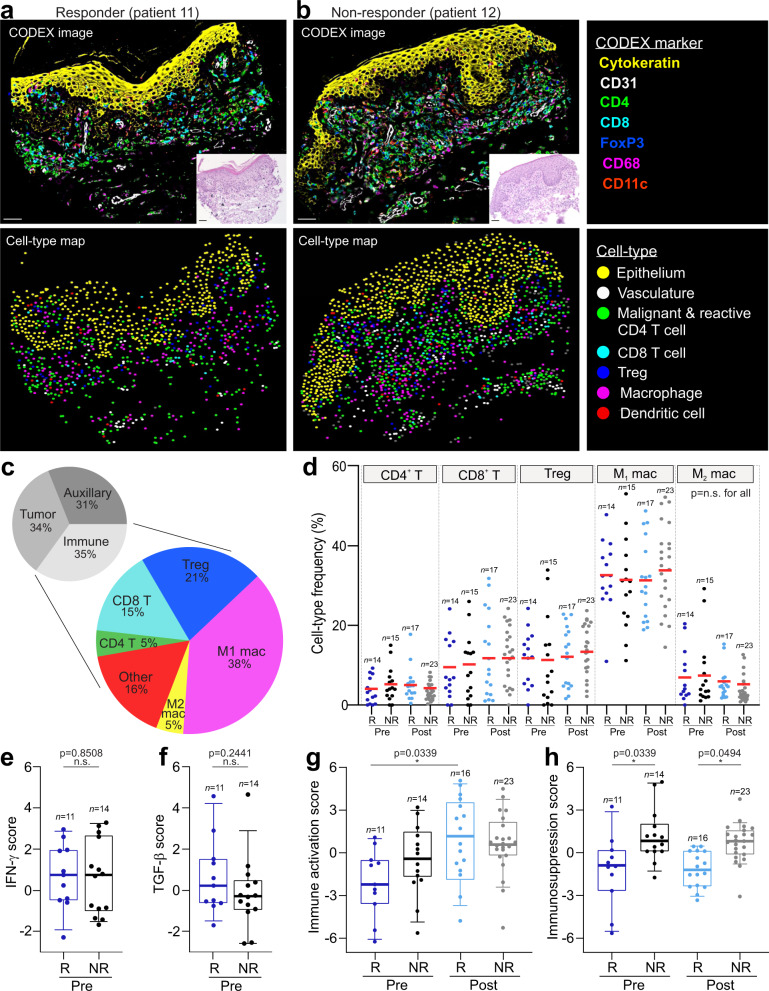
Characterization of the CTCL TME pre- and postpembrolizumab treatment. **a**, **b** Top panels: Representative CODEX seven-color overlay images from a responder (left) and nonresponder (right) pretreatment. Scale bar, 50 µm. Insets, corresponding H&E images; scale bars, 50 µm. Bottom panels: corresponding cell type maps. **c** Upper pie chart: overall frequencies of tumor, immune and auxiliary cell types. Lower pie chart: overall frequencies of all immune cell types, including CD4^+^ T cells, CD8^+^ T cells, Tregs, M1 macrophages, M2 macrophages, and other (B cells, dendritic cells, Langerhans cells, mast cells, neutrophils, and plasma cells). **d** Cell type frequencies of CD4^+^ T cell, CD8^+^ T cell, Treg, M1 macrophage, and M2 macrophage as a percentage of all immune cells per tissue microarray spot across patient groups (mean, red bar). *P* values calculated with a linear mixed-effect model with Bonferroni’s corrections for multiple comparisons (*p* = not significant (n.s.) for all comparisons). **e**, **f** Pretreatment IFN-γ (**e**) and TGF-β (**f**) gene scores per tissue microarray spot in responders and nonresponders. For all box plots: box center line, median; box limits, upper and lower quartiles; box whiskers, 1.5x the interquartile range (IQR). *P* values calculated with a linear mixed-effect model with Bonferroni’s corrections for multiple comparisons. **g**, **h** Immune activation (**g**) and immunosuppression (**h**) gene scores, computed on bulk RNA-seq data, per tissue microarray spot across patient groups. Boxes, median ± upper and lower quartiles; whiskers, 1.5x IQR. *P* values calculated with a linear mixed-effect model with Bonferroni’s corrections for multiple comparisons. Source data are provided as a Source Data file.

To delineate the complex interactions of tumor and immune cells within the TME, we next characterized the cellular composition of CTCL tumors pre- and postpembrolizumab therapy. The 21 cell types identified by CODEX were cataloged across patient groups, including 13 immune cell types, 6 auxiliary cell types, and 2 tumor cell types (Supplementary Fig. 2b). Markers for T cell subsets (CD4, CD8, FoxP3), macrophages (CD68), dendritic cells (CD11c), tumor cells (CD4), vasculature (CD31), and epithelium (cytokeratin) were clearly visualized in the CODEX fluorescent images (Fig. 2a, b, upper panels; Supplementary Fig. 3a). The corresponding hematoxylin and eosin (H&E) images (Fig. 2a, b, inserts; Supplementary Fig. 3b) confirmed accurate staining of structural elements like epithelium and vasculature. Fluorescent staining of immune and tumor cells confirmed the cell type assignments shown in the corresponding cell type maps (Fig. 2a, b, lower panels).

No differences in the cellular composition were noted between responders and nonresponders pre- or post-treatment (Fig. 2c, d; Supplementary Fig. 2c). The combined frequencies of tumor, immune and auxiliary cell types each comprised approximately one-third of all cells (Fig. 2c, upper panel); the same trend held across patient groups (Supplementary Fig. 2c). Among all immune cells, the ranked cell type frequencies were 38% for M_1_ macrophages, 21% for Tregs, 15% for CD8^+^ T cells, 5% for M_2_ macrophages, 5% for CD4^+^ T cells, and <5% for other immune cell types, including B cells, plasma cells, dendritic cells, Langerhans cells, mast cells, and neutrophils (Fig. 2c, lower panel). No differences in the mean frequencies of immune cell types were observed between patient groups; this is highlighted for T cells (CD4^+^ T, CD8^+^ T, Tregs) and macrophages (M1 and M2) (Fig. 2d). This finding is consistent with the full clinical trial cohort^13^ and our baseline IHC data (Fig. 1c, d), which showed no correlation between pembrolizumab response and the expression of T cell, macrophage or PD-1 signaling markers. However, it contrasts with some solid tumor studies, which have correlated the frequency of reactive T cells with clinical outcome^34,35^ and immunotherapy response^36,37^.

We then focused on differences in immune signaling between responders and nonresponders. Immunogenomic analyses were performed to examine the functional immune state of the TME, which has been shown to be a key determinant of immunotherapeutic activity^38^. Gene expression signatures that have predicted PD-1/PD-L1 blockade response (e.g., IFN-γ scores^39^, Supplementary Table 3a) and nonresponse (e.g., TGF-β scores^40^, Supplementary Table 3b) in solid tumors to our CTCL data. No differences were observed between patient groups for the IFN-γ (Fig. 2e) or TGF-β (Fig. 2f) gene scores. While neither signature was predictive of pembrolizumab response in this CTCL cohort, it is important to point out that these gene signatures were derived from single-disease studies, and thus their application may be limited to similar solid tumor types^41^.

Rare cancers like CTCL do not have specifically defined immune gene signatures, making it a challenge to catalog their tumor immunogenicity. Thus, gene lists of immune activation (e.g., *CD27*, *EOMES*, and *ICOS*) and immunosuppression (e.g., *ENTPD1*, *TGFB1*, and *TIGIT*) molecules (Supplementary Table 3c, d) were used to assess the functional immune state of the TME. Genes like *PDCD1* and *TNFRSF18*, which can be immune activating and/or suppressive depending on the cellular state and microenvironmental context, were excluded from this analysis. The immune activation gene score was significantly increased in responders post-treatment compared to pretreatment (Fig. 2g), with no significant change in nonresponders. In contrast, the immunosuppression gene score was significantly increased in nonresponders compared to responders both pre- and post-treatment (Fig. 2h). These findings indicate that 1) nonresponders have an immunosuppressed phenotype relative to responders, 2) only responders develop an immune-activated TME following pembrolizumab treatment, and 3) the functional immune state of the CTCL TME depends on factors beyond raw immune cell counts.

### PD-1 blockade induces spatial re-organization of the CTCL TME

**Fig. 3:**
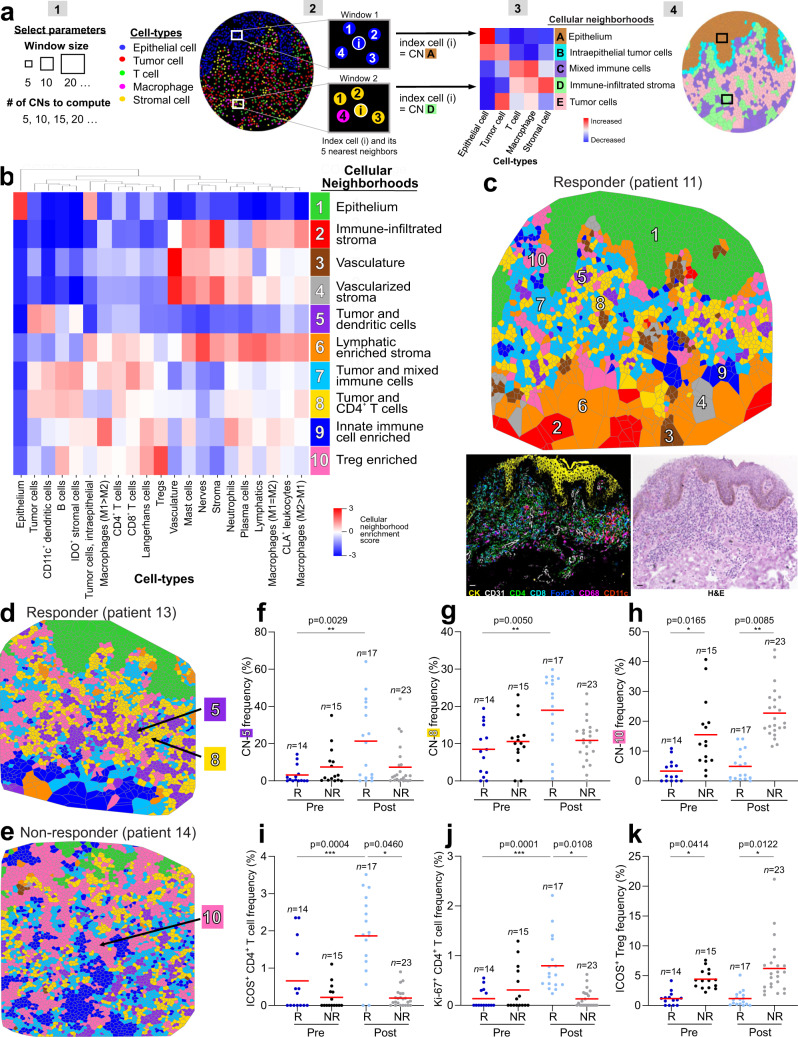
Cellular neighborhoods reveal differences in the spatial TME organization in responders and nonresponders. **a** Cellular neighborhood (CN) analysis schematic. [1] Selection of computational parameters, including the window size (five in this schematic) and the number of CNs to be computed (five in this schematic). [2] Assignment of an index cell (i, center of window) to a given CN based on the composition of cell types within its corresponding window the clustering of windows. [3] Heatmap of cell type distribution for each CN and assignment of CN names. [4] Visualization of CNs as a Voronoi diagram. **b** Identification of 10 conserved CNs in the CTCL TME using a window size of 10. **c** Representative Voronoi diagram of the 10 CNs in a responder post-treatment, with the corresponding H&E and seven color fluorescent CODEX images. Scale bar, 20 µm. **d**–**e** Voronoi diagrams of CNs in a responder (**d**) and nonresponder (**e**) post-treatment, highlighting CN-5 (tumor and dendritic cells), CN-8 (tumor and CD4^+^ T cells) and CN-10 (Treg enriched). **f**–**h** Frequencies of CN-5 (**f**), CN-8 (**g**) and CN-10 (**h**) per tissue microarray spot across patient groups (mean, red bar). *P* values calculated with a linear mixed-effect model with Bonferroni’s corrections for multiple comparisons. **i**–**k** Frequencies of ICOS^+^ CD4^+^ T cell (**i**), Ki-67^+^ CD4^+^ T cell (**j**) and ICOS^+^ Treg (**k**) as a percentage of all immune cells per tissue microarray spot across patient groups (mean, red bar). *P* values calculated with a linear mixed-effect model with Bonferroni’s corrections for multiple comparisons. Source data are provided as a Source Data file.

Anticipating that the functional immune state of the TME was mediated by its spatial cellular organization, we reasoned that specific global tissue patterns would be reflected by a pembrolizumab responsive and nonresponsive phenotype. To obtain a quantitative, high-level view of the CTCL tissue architecture, cellular neighborhood (CN) analysis^15^ was performed. CNs are analogous to urban neighborhoods—essentially geographically localized areas within a larger city that facilitate social interaction^42^ (Supplementary Fig. 2e). Likewise, CNs are defined by a localized enrichment of specific cell types within the tissue that mediate cellular interactions and vital tissue functions (Supplementary Fig. 2f). Computationally, the CN algorithm extracts quantitative data on the composition and spatial distribution of individual cells to reveal how local cellular niches are organized within tissues (Fig. 3a)^15^. Specifically, computational parameters like the window size and number of CNs to be computed are manually set (Fig. 3a.1). Each cell in the tissue is assigned to a given CN based on the composition of cell types within the specified window (Fig. 3a.2). The windows are then clustered and the correlation of CNs and cell types are represented as a heatmap (Fig. 3a.3). CNs are visualized as Voronoi diagrams and analyzed to better understand cellular spatial behavior (Fig. 3a.4). Importantly, these Voroni diagrams account for the spatial interactions between different cell types and as such provide a layer of information beyond the cell type maps shown in Fig. 2a, b.

Using a window size of 10 (i.e., one center cell and its nine nearest neighbors), we identified 10 distinct CNs that were conserved across this CTCL cohort (Fig. 3b, Supplementary Fig. 3c). Some CNs recapitulated structural components that were clearly discernable in the corresponding H&E and fluorescent images, such as epithelium (CN-1, green region) and vasculature (CN-3, brown region) (Fig. 3c). The other CNs were composed of previously unappreciated sub-structures within the dermal infiltrate that were not apparent in the corresponding H&E or fluorescent images, including immune-infiltrated stroma (CN-2, red region), vascularized stroma (CN-4, gray region), tumor and dendritic cells (CN-5, purple region), lymphatic enriched stroma (CN-6, orange region), tumor and mixed immune cells (CN-7, cyan region), tumor and CD4^+^ T cells (CN-8, yellow region), innate immune cell enriched (CN-9, blue region), and Treg enriched (CN-10, pink region) (Fig. 3c).

Comparing representative Voronoi diagrams from a responder (Fig. 3d) and nonresponder (Fig. 3e) showed that the frequencies of three CNs were significantly different between responders and nonresponders. CNs enriched in tumor and dendritic cells (CN-5, purple region; Fig. 3f) and tumor and CD4^+^ T cells (CN-8, yellow region; Fig. 3g) were present at significantly higher frequencies in responders post-treatment compared to other groups. This suggests that the more immune-activated TME observed in responders following pembrolizumab therapy (Fig. 3g) may be mediated by CD4^+^ T cell activation by antigen-presenting cells. Indeed, after PD-1 blockade, responders had increased frequencies of activated ICOS^+^ CD4^+^ T cells (Fig. 3i) and proliferating Ki-67^+^ CD4^+^ T cells (Fig. 3j) compared to responders pretreatment and nonresponders post-treatment, suggesting that pembrolizumab activates CD4^+^ T cells through upregulation of immune-activating molecules.

In contrast, the Treg enriched CN (CN-10, pink region) was present at a significantly higher frequency in nonresponders than responders pre- and post-treatment (Fig. 3h), consistent with our finding that nonresponders had a persistently immunosuppressed TME (Fig. 2h). This finding is further supported by the increased frequencies of a potently suppressive subset of Tregs expressing ICOS (Fig. 3k)^43,44^ and a suppressive subset of IDO-1^+^ CD8^+^ T cells in nonresponders relative to responders (Supplementary Fig. 2g)^45^. Despite treatment with PD-1 blockade, no differences were observed in PD-1^+^ subsets of CD4^+^, CD8^+^, Tregs, or tumor cells between groups (Supplementary Fig. 2h-k).

These data link the spatial organization of tumor and immune cell types with the functional immune state of the TME, highlighting specific patterns of immune control and pembrolizumab responsiveness. Importantly, these spatial interaction differences (i.e., CN-5, CN-8, CN-10) between patient groups occurred even though there were no differences in the abundance of dendritic cells, CD4^+^ T cells, Tregs, or tumor cells (Fig. 2c, d, Supplementary Fig. 4j).

### Spatial signature of PD-1^+^ CD4^+^ T cells, tumor cells, and Tregs predicts pembrolizumab response in CTCL

**Fig. 4:**
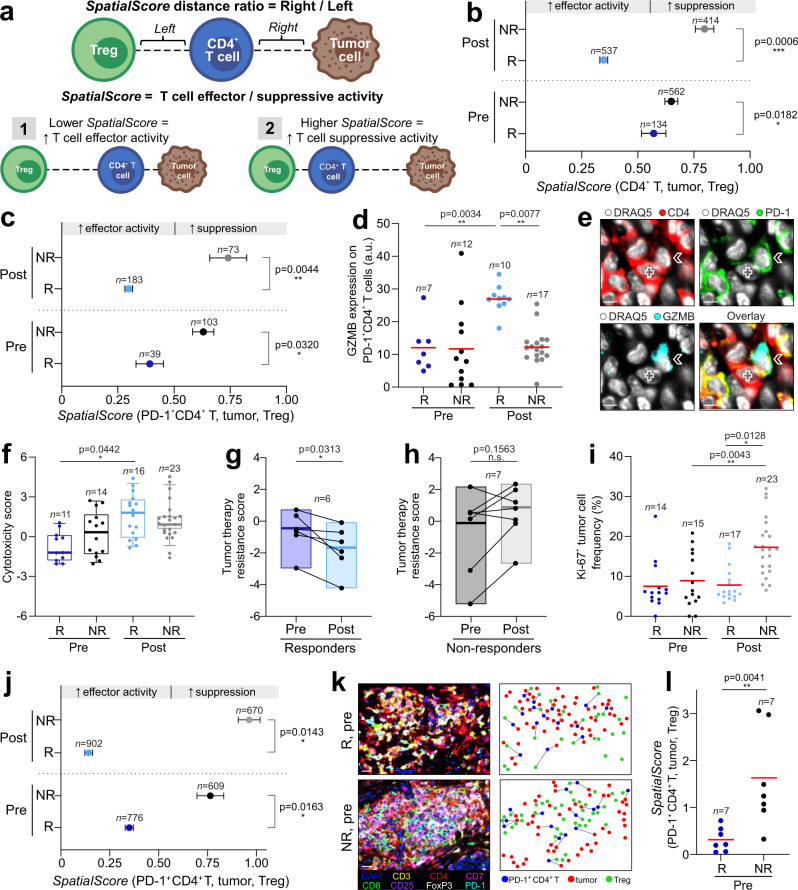
Spatial relationship between CD4^+^ T cells, Tregs and tumor cells predicts pembrolizumab response in CTCL. **a** *SpatialScore* schematic. The *SpatialScore* is calculated by taking the ratio of the physical distance between each CD4^+^ T cell and its nearest tumor cell (distance “right”) relative to its nearest Treg (distance “left”). [1] A lower *SpatialScore* (i.e., CD4^+^ T cells closer to tumor cells than Tregs) suggests increased T cell effector activity. [2] A higher spatial score (i.e., CD4^+^ T cells closer to Tregs than tumor cells) suggests increased T cell suppression. **b**–**c** *SpatialScore* calculated per cell for all CD4^+^ T cells (**b**) and PD-1^+^ CD4^+^ T cells (**c**) across patient groups (mean ± s.e.m.). *P* values calculated with a linear mixed-effect model taking a patient identifier as a random effect. **d** GZMB protein expression on PD-1^+^ CD4^+^ T cells by CODEX per tissue microarray spot (mean fluorescence intensity (arbitrary units, a.u.), red bar). *P* values calculated with a linear mixed-effect model with Bonferroni’s corrections for multiple comparisons. **e** CODEX images showing contact between a tumor cell (cross) and GZMB-expressing PD-1^+^ CD4^+^ T cell (arrow) in responder patient 13 post-treatment. Scale bars, 10 µm. **f** Cytotoxicity gene scores, computed on bulk RNA-seq data, per tissue microarray spot. Boxes, median ± upper and lower quartiles; whiskers, 1.5x IQR. *P* values calculated with a linear mixed-effect model with Bonferroni’s corrections for multiple comparisons. **g**–**h** Pre- to post-treatment changes in tumor therapy resistance gene scores, computed on bulk RNA-seq data, per patient in responders (**g**) and nonresponders (**h**). Boxes, median ± upper and lower quartiles; whiskers, 1.5x IQR. *P* values were calculated by two-sided Wilcoxon’s signed-rank tests. **i** Ki-67^+^ tumor cell frequencies per tissue microarray spot (mean, red bar). *P* values calculated with a linear mixed-effect model with Bonferroni’s corrections for multiple comparisons. **j** *SpatialScore* calculated from Vectra mIHC data per cell for all PD-1^+^CD4^+^ T cells (mean ± s.e.m.). *P* values calculated with a linear mixed-effect model taking a patient identifier as a random effect. **k** Vectra mIHC images (left panels) and corresponding spatial plots (right panels)from responder patient 13 (R) and nonresponder patient 14 (NR) pretreatment. Scale bars, 20 µm. **l** *SpatialScore* calculated from Vectra mIHC data per patient in responders and nonresponders pretreatment(mean, red bar). *P* value calculated by a two-sided Wilcoxon’s rank-sum test, with no adjustments for multiple hypotheses. Source data are provided as a Source Data file.

While complex, computationally intensive spatial analyses have been used to prognosticate cancer outcomes^15,46^, we postulated that a streamlined approach based on the distances between a specific tumor and immune cell types could be employed to identify a predictive biomarker of immunotherapy response. Guided by our CN findings, we formalized a computational scoring approach based on the spatial interactions of CD4^+^ T cells, tumor cells, and Tregs. This score, termed the *SpatialScore*, calculates the physical distance ratio of each CD4^+^ T cell and its nearest tumor cell (“right” distance) relative to its nearest Treg (“left” distance) (Fig. 4a). A lower *SpatialScore* (i.e., CD4^+^ T cells are closer to tumor cells than Tregs) suggests increased T cell effector activity (Fig. 4a.1), whereas a higher *SpatialScore* (i.e., CD4^+^ T cells are closer to Tregs than tumor cells) suggests increased T cell suppression (Fig. 4a.2). As such, the *SpatialScore* can be viewed as a proxy of the balance between T cell effector and immunosuppressive activity in the TME.

The *SpatialScore* was calculated on a per-cell basis (Supplementary Data 4) and the mean value was reported for each patient group. When calculated with all CD4^+^ T cells, the pretreatment *SpatialScore* was significantly lower in responders than nonresponders, with enhancement post-treatment (Fig. 4b, compare mean *SpatialScore* for R, pre (0.57) versus NR, pre (0.63)). The same trend was observed on a per-patient basis (Supplementary Fig. 4a). Since the current study trialed PD-1 blockade, we next asked how the *SpatialScore* was influenced by the PD-1^+^ CD4^+^ T cell subset. As observed with all CD4^+^ T cells, when computed with PD-1^+^ CD4^+^ T cells, tumor cells, and Tregs, the *SpatialScore* was lower in responders than nonresponders (Fig. 4c, compare mean *SpatialScore* for R, pre (0.40) versus NR, pre (0.62)). The same trend was seen on a per-patient basis (Supplementary Fig. 4b). Interestingly, the *SpatialScore* was lower in responders pretreatment when calculated with PD-1^+^ CD4^+^ T cells versus all CD4^+^ T cells, implying increased effector activity in this T cell subset. These results suggest that PD-1^+^ CD4^+^ T cells are primed for increased antitumor activity in responders, which is enhanced in the immune-activated TME that develops following pembrolizumab therapy.

As the *SpatialScore* appears to predict the outcome of PD-1 blockade in CTCL, it stands to reason that there is a deep phenotype of cell type-specific architecture that is driving this spatial biomarker. Pretreatment differences were driven by the closer proximity of PD-1^+^ CD4^+^ T cells and Tregs in nonresponders (Supplementary Fig. 4c, see red arrow), consistent with the increased immunosuppression gene scores in nonresponders relative to responders (Fig. 2h). In contrast, post-treatment differences were driven by the closer proximity of PD-1^+^ CD4^+^ T cells and tumor cells in responders (Supplementary Fig. 4d, see red arrow), consistent with the increased immune activation gene score in responders relative to nonresponders (Fig. 2g).

Importantly, no correlation was identified between the abundance of PD-1^+^ CD4^+^ T cells, tumor cells, or Tregs and the *SpatialScore* per tissue microarray spot (Supplementary Fig. 4g–i), showing that the *SpatialScore* is not merely driven by cell type frequency. Additionally, the mean *SpatialScore* was significantly different from that of a random sample in responders (Supplementary Fig. 4k, Supplementary Fig. 4m), but not in nonresponders (Supplementary Fig. 4l, Supplementary Fig. 4n), suggesting that an active process specifically coordinates the spatial interactions of PD-1^+^ CD4^+^ T cells, tumor cells, and Tregs in responders. Finally, when the *SpatialScore* was calculated for CD8^+^ T cells (Supplementary Fig. 4e) and PD-1^+^ CD8^+^ T cells (Supplementary Fig. 4f), it was not predictive of therapeutic response. Collectively, these findings suggest that CD4^+^ T cells and the PD-1^+^ CD4^+^ T cell subset may have a more important effector function in CTCL than previously appreciated, in line with prior observations in Hodgkin lymphoma^47^.

### PD-1^+^ CD4^+^ T cells upregulate cytotoxic effector molecules in pembrolizumab responders

To further characterize the mechanisms contributing to the *SpatialScore*, we used CODEX and RNA-seq datasets to explore whether the closer proximity of PD-1^+^ CD4^+^ T cells and tumor cells in responders reflected enhanced T cell effector function. Among all granzyme B (GZMB) positive immune cells, 8.4% were CD4^+^ T cells. In CTCL, cytotoxic CD4^+^ T cells act through a granzyme-perforin-dependent pathway to achieve tumor cell killing^48,49^, similar to cytotoxic CD8^+^ T cells^49–51^. The expression of GZMB on PD-1^+^ CD4^+^ T cells was increased in responders post-treatment relative to other patient groups (Fig. 4d). This increased GZMB expression was confirmed visually with DRAQ5 (nuclear stain), CD4, PD-1, and GZMB fluorescent staining (Fig. 4e, see white arrow) and was consistent with the increased cytotoxicity gene score^52,53^ seen in responders post-treatment (Fig. 4f, Supplementary Table 3e). No such cytotoxicity was observed for PD-1^+^ CD4^+^ T cells in nonresponders (Fig. 4d, e).

We also postulated that increased cytotoxicity would coincide with a decreased resistance of tumor cells to therapy. Using genes associated with a poor treatment response^54^ and progressive^55^ CTCL (Supplementary Table 3f), it was observed that tumor cell resistance to pembrolizumab decreased in 100% of responders (Fig. 4g). In contrast, 71% of nonresponders had an increased resistance to immunotherapy (Fig. 4h) and an increased frequency of proliferating (Ki-67^+^) tumor cells following treatment (Fig. 4i), consistent with the development of progressive disease in a subset of nonresponders following PD-1 blockade^13,26,56^. Collectively, these data show that responders have the superior cytotoxic potential of their effector PD-1^+^ CD4^+^ T cells following pembrolizumab therapy compared to nonresponders, which coincides with the post-treatment enhancement of the S*patialScore*.

### Validation of the *SpatialScore* with a clinically accessible multiplexed IHC platform

Although highly multiplexed spatial approaches like CODEX provide the raw data for deep cellular profiling and biomarker discovery, translating these findings to a clinical arena requires simplifying the predictive spatial signature to a diagnostic platform that can be readily implemented in clinical practice. Vectra, in conjugation with the Phenoptics workflow, is a commercially available, widely adopted clinical mIHC imaging platform, which has been used to identify biomarkers for renal cell carcinoma^57^ and B cell lymphoma^58^. We devised a simplified staining panel (DAPI, CD3, CD4, CD7, CD8, CD25, FoxP3, and PD-1) that captured the PD-1^+^ CD4^+^ T cells, tumor cells, and Tregs used to calculate the *SpatialScore*. Notably, the tumor cell phenotype identified by CODEX in this cohort (i.e., decreased expression of CD7 and increased expression of CD25 and Ki-67; see Fig. 1h), was critical for establishing this condensed staining panel and was readily transferred to the Vectra platform.

Serial sections from the same CTCL TMA used for CODEX and RNA-seq were stained with this simplified panel and imaged with Vectra (Supplementary Fig. 5a). Across all TMA spots, 126,653 cells were identified, including 2957 PD-1^+^ CD4^+^ T cells, 6161 Tregs, and 19,847 tumor cells. The *SpatialScore* was then computed on a per-cell basis (Supplementary Data 5) and the mean was reported for each patient group. Consistent with the CODEX results (Fig. 4c), the Vectra-derived *SpatialScore* was significantly lower in responders than nonresponders pretreatment (Fig. 4j, mean *SpatialScore* R, pre (0.35) versus NR, pre (0.76)), with enhancement post-treatment compared to pretreatment. The Vectra mIHC images (Fig. 4k, left) and corresponding spatial maps (Fig. 4k, right) provide visual validation at the single-cell level that PD-1^+^ CD4^+^ T cells were closer to tumor cells in responders but closer to Tregs in nonresponders. Furthermore, on a per-patient basis, the pretreatment *SpatialScore* was nearly five-times lower in responders than nonresponders (Fig. 4l, mean *SpatialScore* R, pre (0.31) versus NR, pre (1.52)), with excellent biomarker performance measures at a *SpatialScore* cutoff point of 0.7908 (Supplementary Fig. 5b–e). When calculated per patient, the *SpatialScore* extended over a wider range than when calculated for all cells per group; this is due to sample size differences (i.e., *n* = 7 per patient versus *n* = 1000 s of cells per group). Collectively, these results show that findings identified by highly multiplexed imaging platforms like CODEX can be translated to more clinically accessible platforms like Vectra. This is critical for implementing the *SpatialScore* concept to identify biomarkers of immunotherapy response across tumor types.

Although the patient cohort studied herein (*n* = 14 patients) is comparable to other molecular CTCL studies (*n* = 1–14 patients)^9,31,59–64^, we performed subsampling and patient-exclusion analyses to validate that our sample size was sufficient to detect clinically relevant and statistically robust differences in the *SpatialScore*. First, 100 iterations of subsampling were performed at two independent values^65^; namely 50 or 75% of the Vectra-derived pretreatment *SpatialScore* data (*n* = 1385 *SpatialScore* measurements). We next tested whether the mean *SpatialScore* was significantly different between responders and nonresponders within each iteration of the sampled data. The distribution of *p* values for the mean *SpatialScore* differences between patient groups for each of these 100 iterations shows that even with 50% data subsampling, most iterations yield a *p* value < 0.05 (Supplementary Fig. 5f; the proportion of statistically significant *p* values is 73 and 98% for subsamples of 50 and 75%, respectively). Estimation statistics is a simple visual framework to ascertain different effect sizes between compared groups, displaying all data points and its effect size distribution as a bootstrap 95% confidence interval^66,67^. Visualizing the mean *SpatialScore* for each iteration with bootstrap-coupled estimation^67^, the mean *SpatialScore* difference between paired responders and nonresponders (i.e., effect size) across 100 subsampling iterations of 50% (Supplementary Fig. 5g) and 75% (Supplementary Fig. 5h) of the data was statistically significant. To further confirm the robustness of our findings, we performed exclusion analysis on the Vectra-derived *SpatialScore* data. For this analysis, we iteratively excluded one of the 14 patients and confirmed that the *SpatialScore* between responders and nonresponders remained statistically significant overall 14 iterations (Supplementary Fig. 5i). Furthermore, bootstrap-coupled estimation of the mean *SpatialScore* differences between responders and nonresponders across these 14 iterations of patient exclusion confirmed a large effect size between the two patient groups (Supplementary Fig. 5j). These statistical analyses show that a sample size of 14 patients was sufficient to stratify responders and nonresponders based on the *SpatialScore*.

### Tumor cell-specific CXCL13 expression coincides with a favorable response to PD-1 blockade

**Fig. 5:**
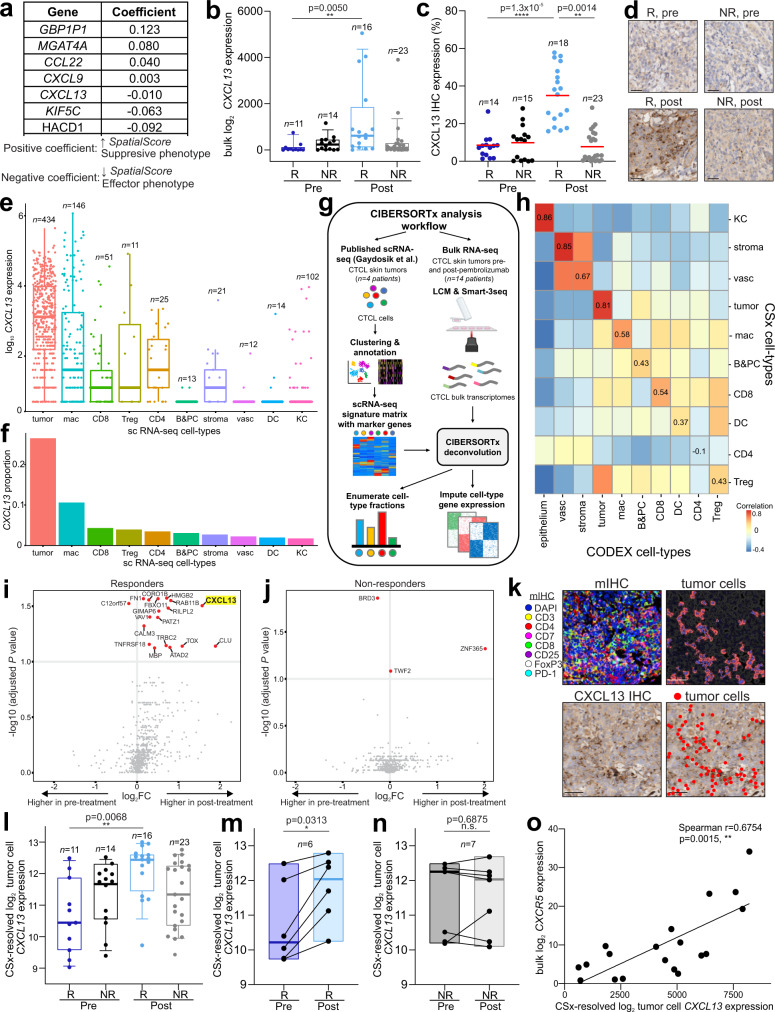
CXCL13 is a key driver of pembrolizumab response in CTCL. **a** Seven genes from bulk RNAseq data predictive of the *SpatialScore*. **b** Normalized bulk *CXCL13* gene expression per tissue microarray spot. Boxes, median ± upper and lower quartiles; whiskers, 1.5x IQR. *P* values calculated with a linear mixed-effect model with Bonferroni’s corrections for multiple comparisons. **c** CXCL13 protein expression by IHC per tissue microarray spot (mean, red bar). *P* values calculated with a linear mixed-effect model with Bonferroni’s corrections for multiple comparisons. **d** Representative CXCL13 IHC images from responder patient 9 (left panels) and nonresponder patient 14 (right panels). Scale bars, 20 µm. **e**–**f** *CXCL13* expression in single-cell transcriptomes from CTCL skin tumors (Gaydosik et al.)^62^. **e** Normalized expression of *CXCL13* in single cells; excluded cells with *CXCL13* log1p normalized read counts < 0.5. Boxes, median ± upper and lower quartiles; whiskers, 1.5x IQR. **f** Proportion of CXCL13-expressing cells per cell type. **g** CIBERSORTx workflow schematic. A CSx deconvolution signature matrix was generated from single-cell transcriptomes (Gaydosik et al.)^62^ (left) and applied to CTCL bulk transcriptomes obtained with laser-capture microdissection (LCM) and Smart-3Seq (right) to enumerate cell type fractions and resolve gene expression profiles. **h** Heatmap correlation of CSx-resolved and CODEX-identified cell type frequencies; Spearman coefficients are on the diagonal. **i**–**j** Differential expression of CSx-resolved tumor cell genes in responders (**j**) and nonresponders (**k**) pre- and post-treatment. *P* values calculated with a linear mixed-effect model with Benjamini-Hochberg correction (significantly different genes (*p* < 0.1), red; *CXCL13* highlighted yellow). **k** Vectra mIHC image (top left), corresponding tumor cell depiction (top right), corresponding CXCL13 IHC image (bottom left), and corresponding overlay image of CXCL13 staining and tumor cells (bottom right) in responder patient 9 post-treatment. Scale bars, 20 µm. **l** Normalized CSx-resolved *CXCL13* expression in tumor cells per tissue microarray spot. Boxes, median ± upper and lower quartiles; whiskers, 1.5x IQR. *P* values calculated with a linear mixed-effect model with Bonferroni’s corrections for multiple comparisons. **m**–**n** Pre- to post-treatment changes in normalized *CXCL13* gene expression from CSx-resolved tumor genes per patient in responders (**m**) and nonresponders (**n**). Boxes, median ± upper and lower quartiles; whiskers, 1.5x IQR. *P* values calculated by two-sided Wilcoxon’s signed-rank tests. **o** Correlation of CSx-resolved tumor cell *CXCL13* expression and bulk *CXCR5* expression per tissue microarray spot. Data evaluated with two-sided Spearman test. Source data are provided as a Source Data file.

Finally, to add insight into the complex molecular processes driving the *SpatialScore*, we examined potential recruitment mechanisms by identifying genes predictive of the spatial interactions between PD-1^+^ CD4^+^ T cells, tumor cells, and Tregs. An L1-regularized linear model was fit to bulk RNA-seq data on a per spot basis, which revealed seven genes predictive of the *SpatialScore* including three chemokines: *CXCL9*, *CCL22*, and *CXCL13* (Fig. 5a). *CXCL9* and *CCL22* are known to mediate Treg recruitment^68,69^; they had positive coefficients and were predictive of the higher *SpatialScore* seen in nonresponders. *CXCL13* is a chemoattractant expressed on benign lymphocytes and CTCL tumor cells^28,70^; it had a negative coefficient and was predictive of the lower *SpatialScore* seen in responders. Bulk *CXCL13* gene expression was significantly increased in responders post-treatment compared to other patient groups (Fig. 5b), which was confirmed by *CXCL13* IHC shown quantitatively (Fig. 5c) and visually (Fig. 5d).

We then analyzed a publicly available scRNA-seq dataset of CTCL skin tumors^62^ to identify the main cell type overexpressing *CXCL13*. Ten clusters were annotated including tumor cells, CD4^+^ T cells (CD4), Tregs, CD8^+^ T cells (CD8), B & plasma cells (B&PC), macrophages (mac), dendritic cells (DC), stroma, vasculature (vasc), and keratinocytes (KC) (Supplementary Fig. 6a, b). All cells with a *CXCL13* log1p normalized read count >0.5 were analyzed and tumor cells had the highest mean expression of *CXCL13* (Fig. 5e). Additionally, the frequency of CXCL13-expressing cells was highest among tumor cells compared to other cell types (Fig. 5f).

CIBERSORTx (CSx) is a computational framework that uses gene expression signatures to enable cell type-specific gene expression to be inferred from bulk RNA-seq data without physical cell isolation^21^. We used CSx to computationally resolve tumor, stromal, and immune cell subsets in bulk RNA-seq data (Fig. 5g, Supplementary Table 4a, b, Supplementary Data 3, Supplementary Data 6). Using the 10 annotated clusters shown in Fig. 5e, f, and Supplementary Data 7, a signature matrix, consisting of cell type-specific marker genes, was generated (Supplementary Fig. 6c, Supplementary Data 8, see Methods). This signature matrix was used to enumerate CSx-resolved cell type fractions and resolve gene expression profiles from CTCL bulk transcriptomes. Strong correlations were observed between the CSx-resolved cell type frequencies and CODEX-identified cell type frequencies (Fig. 5h, Supplementary Fig. 7, Supplementary Fig. 8), confirming the utility of the CSx approach.

The CSx-resolved gene expression profiles of tumor cells were thoroughly characterized and compared across patient groups. Interestingly, *RAB11B*, a RAS superfamily member of small GTP-binding proteins, was the only tumor cell gene with significantly different expression between responders and nonresponders pretreatment (Supplementary Fig. 6d), suggesting an absence of intrinsic tumor cell differences between patient groups at baseline. In contrast, numerous genes, including *CXCL13*, were upregulated in the tumor cells of responders post-treatment compared to pretreatment (Fig. 5i, see *CXCL13* in bold). This finding is consistent with an increased susceptibility of responder tumor cells to PD-1 blockade therapy and supported by the decreased tumor therapy resistance score observed in responders post- vs pretreatment (Fig. 4g). Interestingly, only three genes (*BRD3*, *TWF2*, and *ZNF365*) were differentially expressed in nonresponder tumor cells post-treatment compared to pretreatment (Fig. 5j), which suggests that nonresponder tumor cells are resistant to PD-1 blockade, in line with the increased tumor therapy resistance score observed in nonresponders post- vs pretreatment (Fig. 4h). Finally, co-staining the tissue section used for the Vectra mIHC experiment with an anti-CXCL13 antibody by standard IHC, followed by co-localization analysis of tumor cells and CXCL13-positive cells, provided visual confirmation that tumor cells are the primary expressors of CXCL13 (Fig. 5k, Supplementary Fig. 6e).

Next, we assessed *CXCL13* expression in tumor cells and its role in recruiting reactive lymphocytes. CSx-resolved tumor cell-specific *CXCL13* expression was significantly increased in responders post-treatment compared with other patient groups on a per tissue microarray spot basis (Fig. 5l). On a per-patient basis, tumor cell-resolved *CXCL13* expression increased in 100% of responders post- vs pretreatment (Fig. 5m) versus 29% of nonresponders (Fig. 5n). CXCL13 exclusively binds to the chemokine receptor CXCR5, which is expressed on B cells, CD4^+^ T cells, CD8^+^ T cells, and skin-derived migratory dendritic cells^71^. *CXCR5* expression was increased in bulk mRNA of responders post-treatment relative to pretreatment, but did not reach statistical significance (Supplementary Fig. 6f). The data lacked significant power to unmix *CXCR5* on CD4^+^ T cells by CSx; however, tumor cell-specific *CXCL13* expression was positively correlated with bulk mRNA *CXCR5* expression (Fig. 5o) and a strong receptor-ligand interaction was predicted between *CXCL13* in tumor cells and *CXCR5* in CD4^+^ T cells (Supplementary Fig. 8d), suggesting a chemoattractant recruitment mechanism.

**Fig. 6:**
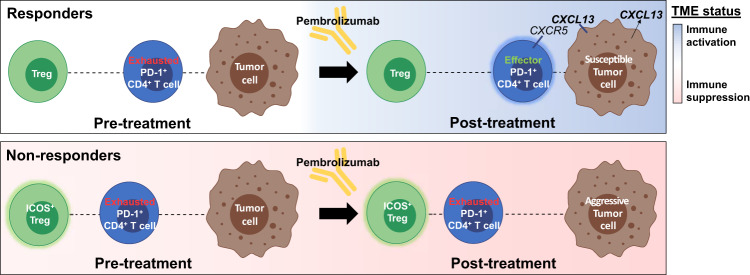
Proposed mechanisms of pembrolizumab response in CTCL. Cartoon depicting proposed mechanisms of pembrolizumab response in therapy responders (top panel) and nonresponders (bottom panel) pre- and post-treatment. The functional immune status of the TME is represented by blue shading when activated and pink shading when suppressed. In nonresponders, the TME is continually immunosuppressed and persistently exhausted PD-1^+^CD4^+^ T cells are in closer proximity to potently suppressive ICOS^+^ Tregs. Moreover, the tumor cells of nonresponders are less susceptible to pembrolizumab therapy. In contrast, responders have a neutral functional immune state pretreatment, which facilitates the baseline spatial organization that underlies a lower *SpatialScore* and immune cell priming and activation. This enables the transition from exhausted PD-1^+^ CD4^+^ T cells to effector/cytotoxic GZMB^hi^ PD-1^+^ CD4^+^ T cells following pembrolizumab therapy. Additionally, responder tumor cells are susceptible to PD-1 blockade and overexpress CXCL13 post-treatment, which chemoattracts effector PD-1^+^ CD4^+^ T cells toward tumor cells, providing a mechanism for the sustained clinical response seen in responders.

Collectively, these results indicate that PD-1 blockade distinctly alters the CTCL TME of therapeutic responders and nonresponders. Pretreatment differences in the functional immune state of responders and nonresponders likely regulate the baseline spatial organization that underlies the *SpatialScore* and the potential for immune cell priming. In responders, pembrolizumab therapy appears to promote T cell activation and upregulate CXCL13 in tumor cells (Fig. 6, top panel). In turn, CXCL13 overexpression seems to attract effector/cytotoxic PD-1^+^ CD4^+^ T cells to tumor cells, which promotes CD4^+^ T cell-mediated tumor cell inhibition and killing. As such, the overexpression of CXCL13 in tumor cells provides a potential mechanism for the sustained clinical activity seen in responders. In contrast, nonresponders have a persistently immunosuppressed TME, which appears to mediate the increased interaction between the inhibitory Tregs and PD-1^+^ CD4^+^ T cells and maintains this CD4^+^ T cell subset in an exhausted state (Fig. 6, bottom panel). In the setting of pembrolizumab resistance, nonresponder tumor cells seem to remain aggressive and proliferative following treatment, accounting for the development of progressive disease. Thus, underlying differences in the functional immune state of the TME—coupled with alterations in CD4^+^ T cell effector activity versus suppression and tumor cell-specific CXCL13 expression—appear to be associated with distinct spatial cellular patterns that predict the efficacy of PD-1 blockade in CTCL.

## Discussion

For anti-PD-1 immunotherapies to provide maximal benefit to cancer patients, the drivers and resistors of clinical response must be identified. Traditional biomarker studies with IHC, gene expression profiling, and tumor mutational burden assays do not fully account for spatial cellular context and have an imperfect correlation with immunotherapy outcomes^72^. This was true for our CTCL cohort: No pretreatment differences were observed in immune composition, expression of PD-1/PD-L1 proteins, or IFN-γ/TGF-β gene signatures between responders and nonresponders. We therefore explored alternative approaches, including spatially-resolved multiplexed tissue imaging, which has been shown to significantly improve the accuracy of predicting response to PD-1 blockade in several tumor types^73^.

CODEX analysis revealed global prognostic spatial patterns that were predictive of clinical response, including a localized enrichment of tumor and CD4^+^ T cells (CN-8) in responders and of Tregs (CN-10) in nonresponders. Profiling the spatial relationships between effector PD-1^+^ CD4^+^ T cells, tumor cells, and immunosuppressive Tregs allowed us to derive the *SpatialScore*—a clinically useful biomarker that correlated strongly with pembrolizumab response in CTCL. While previously identified spatial biomarkers rely on pairwise distances (e.g., PD-1^+^ T cells and PD-L1^+^ tumor cells)^47,74,75^, the *SpatialScore* accounts for the interactions between three functionally distinct cell types. As such, the *SpatialScore* represents a predictive approach and accounts for three key therapeutic determinants underlying PD-1 blockade in CTCL: 1) functional immune state of the TME, 2) T cell function, and 3) chemoattraction.

First, the *SpatialScore* reflects differences in the functional immune phenotypes between CTCL responders and nonresponders. Following pembrolizumab therapy, the TME of responders becomes activated, as evidenced by the increased immune activation gene score, increased frequency of ICOS^+^ and Ki-67^+^ CD4^+^ T cells, and a local enrichment of tumor cells with dendritic cells (CN-5) and CD4^+^ T cells (CN-8). These findings suggest that in responders PD-1 blockade specifically activates CD4^+^ T cells through the expression of immunostimulatory molecules and co-stimulation by antigen-presenting cells, resulting in CD4^+^ T cell proliferation^76,77^. In contrast, nonresponders have a persistently immunosuppressed TME. This is supported by their increased immunosuppression gene score, increased frequency of a Treg enriched neighborhood (CN-10), and the expansion of a highly suppressive ICOS^+^ Treg subset, which has been associated with poor clinical outcomes in melanoma^43^ and liver cancer^44^. These results suggest that both the increased suppressive function and spatial organization of Tregs in nonresponders contribute to the lack of pembrolizumab response. We speculate that the absence of this pretreatment immunosuppression in responders promotes the 1) advantageous spatial interactions of tumor and PD-1^+^ CD4^+^ T cells that underlies the lower *SpatialScore* and 2) priming and activation of CD4^+^ T cells following immunotherapy.

Second, the *SpatialScore* captures differences in T cell function between CTCL responders and nonresponders. After PD-1 blockade, T cell effector activity is restored in responders, whereas nonresponders have a continually exhausted T cell phenotype. Consistent with studies of Hodgkin lymphoma^47^, bladder cancer^53^, and glioblastoma^78^, our data suggest that CD4^+^ T cells, and particularly the PD-1^+^ CD4^+^ T cell subset, are crucial effectors that influence pembrolizumab response in CTCL. In responders post-treatment, PD-1^+^ CD4^+^ T cells increase GZMB expression and move closer to tumor cells, similar to the granzyme-perforin-dependent tumor-killing mechanism used by cytotoxic CD8^+^ T cells^48–51^. Notably, prior studies have shown that antitumor activity is enhanced by a closer proximity of cytotoxic T cells to tumor cells^79,80^ and by removing inhibitory Tregs from the TME milleu^53^. The *SpatialScore* combines these principles by measuring the physical distances between 1) PD-1^+^ CD4^+^ T cells and tumor cells (i.e., effector function) and 2) PD-1^+^ CD4^+^ T cells and Tregs (i.e., suppressive function). The *SpatialScore* therefore reflects the balance of T cell effector versus suppressive activity in the TME, which are key determinants of the efficacy of PD-1 blockade.

Third, increased expression of *CXCL13* is predictive of the lower *SpatialScore* and improved clinical outcomes seen in CTCL responders, consistent with prior studies in the breast^81^, colon^82^, ovarian^83,84^, lung^85^, and urothelial cancers^86^ as well as responsiveness to anti-PD-1 immunotherapy^84,85,87^. We speculate that the increased RNA and protein expression of CXCL13 in responder tumor cells following pembrolizumab therapy is advantageous in localizing effector PD-1^+^ CD4^+^ T cells within the TME. Previous studies show that upregulation of *CXCL13* strongly attracts CXCR5^+^ CD4^+^ T cells to the tumor site^85^ and PD-1^+^ CXCR5^−^ CD4^+^ T cells in IgG4-related disease^88^. Additionally, a recent bladder cancer study showed that the tumor-specific gene expression program of cytotoxic GZMB^+^ CD4^+^ T cells treated with anti-PD-L1 therapy was marked by tumor overexpression of *CXCL13*^53^. Furthermore, CXCL13 null mice with bladder tumors did not respond to anti-PD-1 treatment and had a lower frequency of T cell infiltration compared their wild-type counterparts^86^. Collectively, these findings support a chemoattractant mechanism for the sustained clinical response to pembrolizumab therapy observed in CTCL responders: upregulation of CXCL13 in tumor cells attracts effector PD-1^+^ CD4^+^ T cells, promoting successful antitumor activity. This aspect of pembrolizumab responsiveness is captured by the lower *SpatialScore* seen in responders and underscores the importance of T cell topography as a spatial biomarker.

Presentation of the *SpatialScore* approach provides an important conceptual foundation for identifying spatial biomarkers of immunotherapy response and deeply characterizing the TME. By distilling high-dimensional pathology into a simplified spatial metric that can be routinely measured in clinical practice, the *SpatialScore* represents a major advancement for the clinical use of immunotherapies. However, key questions still need to be addressed: 1) Does this spatial biomarker of PD-1^+^ CD4^+^ T cells, tumor cells, and Tregs translate broadly for the prediction of pembrolizumab response in CTCL?, 2) What is the threshold value for the *SpatialScore* that can be used to clinically stratify CTCL patients into probabilistic responders and nonresponders?, 3) Can scRNA-seq and T cell receptor sequencing studies reveal unique features of PD-1 + CD4 + T cells in CTCL responders and ICOS + Tregs in nonresponders?, 4) Can CXCL13 expression levels—in both skin and blood—serve as surrogates of pembrolizumab activity in CTCL and guide ongoing treatment decisions?, and 5) Can the *SpatialScore* concept (i.e., simplified spatial metric of effector vs immunosuppressive activity in the TME) be translated to other immunotherapies and tumor types? We anticipate that the *SptialScore* paradigm will be adopted by users of highly multiplexed imaging technologies (e.g., CODEX, MIBI, and IMC) and clinically accessible mIHC platforms (e.g., Vectra) alike, which will advance the mechanistic insights of immunotherapy and better inform their clinical use in cancer patients.

## Methods

### Human subjects and clinical trial study design

The CITN-10 trial (NCT02243579) was a multicenter, phase II, single-arm clinical trial that investigated the efficacy of pembrolizumab in 24 patients with two common forms of relapsed/refractory CTCL: mycosis fungoides and Sézary syndrome^13^. Written informed consent was obtained from all enrolled patients in accordance with the latest version of the Declaration of Helsinki. The study protocol and collection of tissues were approved by the Institutional Review Board (IRB) at Stanford University (Protocol #5538). The use of tissues for research was fully de-personalized and approved by the Stanford University IRB Administrative Panels on Human Subjects in Medical Research (HSR #46894). All patients had a clinicopathologically confirmed diagnosis of mycosis fungoides or Sézary syndrome (clinical stage IB to IV) that had relapsed, was refractory to, or had progressed after at least one standard systemic therapy. Exclusion criteria included an age less than 18 years, central nervous system disease, active autoimmune disease, previous exposure to any anti-PD-1, anti-PD-L1, or anti-PD-L2 therapy, or treatment with radiotherapy or other anticancer agents within 15 weeks of the pretreatment biopsy. Topical medications were not applied to the biopsied areas during treatment or within 8 weeks of the pretreatment biopsy. Patients were treated with 2 mg/kg pembrolizumab by intravenous infusion every 3 weeks for up to 24 months^13^. Response and primary endpoint (overall response rate) were assessed by consensus global response criteria^22^.

### Sample collection and tissue microarray construction

Skin biopsy specimens were collected from the primary tumor site and FFPE histology blocks were generated according to standard pathology procedures. Pretreatment biopsies were obtained prior to the first pembrolizumab infusion and post-treatment biopsies were collected during and at the conclusion of therapy (Supplementary Fig. 1, Supplementary Table 1a-b). H&E-stained sections from all biopsies were reviewed by two board-certified pathologists (C.M.S. and R.H.P.). Fourteen of the 24 biopsy samples had adequate FFPE material to analyze. These samples included seven responders (one complete response, six partial response) and seven nonresponders (three progressive disease, four stable disease). Two to three cores of 0.6 mm diameter from the most grossly infiltrated regions of each biopsy sample were digitally annotated and compiled into a tissue microarray. The most infiltrated regions were used to avoid samples that were primarily composed of dermal collagen void of tumor and immune cell types. The tissue microarray was sectioned at 4-µm thickness and mounted onto Vectabond^TM^-treated (Vector Labs, #SP-1800) square glass coverslips (22 × 22 mm, #1 1/2, Electron Microscopy Sciences, #72204-01).

### Immunohistochemistry

IHC for CD3 (clone CD3-12; Abd Serotec), CD4 (clone 4B12; Leica), CD8 (clone CD8/144B; Dako), FoxP3 (clone 236 A/E7; Abcam), CD163 (clone 10D6; Thermo Fisher Scientific), PD-1 (clone NAT105; Cell Marque), PD-L1 (clone 22C3; Merck Research Laboratories), and PD-L2 (clone 3G2; Merck Research Laboratories) was performed as previously described^89^. Images were scored by CITN pathologists, and the positive percentage of the total mononuclear cell infiltrate was reported^13^.

### Multiplex immunohistochemistry and analysis

mIHC was performed as previously described^90^. Briefly, 4-µm FFPE tissue sections were baked for 1 h at 60 °C, dewaxed, and stained on a BOND Rx autostainer (Leica) according to Opal Multiplex IHC assay (Akoya Biosciences)s protocol with the following changes: additional high stringency washes were performed after the secondary antibody and Opal fluor applications using high-salt TBST (0.05 M Tris, 0.3 M NaCl and 0.1% Tween-20, pH 7.2–7.6), and TCT was used as the blocking buffer (0.05 M Tris, 0.15 M NaCl, 0.25% Casein, 0.1% Tween 20, pH 7.6 ± 0.1). The antibody panel was stained in the following order, with antibody stripping between positions. Each primary antibody was incubated for 60 min, followed by 10-min incubation with a secondary antibody (OPAL polymer HRP mouse plus rabbit, Akoya Biosciences), followed by the application of the tertiary TSA-amplification reagent (OPAL Fluor, Akoya Biosciences) for 10 min. Positions were as follows: position 1: CD8 (clone CD8/144B, DAKO #M7103; working concentration 0.8 µg/ml; OPAL Fluor 520); position 2: CD25 (clone 4C9, Cell Marque #125M-14; working concentration 0.17 µg/ml; OPAL Fluor 540); position 3: CD3 (clone SP7, Thermo Fisher Scientific #RM-9107; working concentration 0.06 µg/ml; OPAL Fluor 570); position 4: PD-1 (clone EPR4877(2), Abcam #ab137132, working concentration 1.0 µg/ml; OPAL Fluor 650); position 5: CD7 (clone MRQ-56; Cell Marque #107M-24; working concentration 1.18 µg/ml; OPAL Fluor 690); position 6: CD4 (clone EP204, Epitomics; #AC0173A; working concentration 0.08 µg/ml; OPAL Fluor 480); and position 7: FoxP3 (clone 236 A/E7; eBioscience #14-4777-82; working concentration 5.0 µg/ml; OPAL Fluor 620). Subsequently, slides were stained with Spectral DAPI (Akoya Biosciences) for 5 min, rinsed, and mounted with Prolong Gold Antifade reagent (Thermo Fisher Scientific #P36930). After curing for 24 h at room temperature in the dark, images were acquired on a Vectra Polaris automated quantitative pathology imaging system (Akoya Biosciences). The raw images were spectrally unmixed using the Phenoptics inForm software (Akoya Biosciences) and exported as multi-image TIFF files. These antibodies have been previously validated^90,91^. The staining order and pattern of these antibodies were confirmed in tonsil tissue before proceeding with the CTCL tissue microarray experiment.

After fluorescent imaging, the slides were de-coverslipped, loaded onto the BOND Rx autostainer, stripped of bound antibody, and a post-mIHC staining for CXCL13 (goat polyclonal, R&D Systems #AF801; working concentration 0.5 µg/ml; incubation 60 min) was performed. Bound antibody was revealed by antigoat HRP secondary ImmPress HRP (Vector Labs #MP-7405; concentration ready-to-use; incubation 12 min), followed by DAB chromogen using the BOND Polymer Refine Detection kit (Leica) according to the manufacturer’s instructions. After counterstaining with hematoxylin, slides were dry-mounted and scanned on an Aperio AT turbo digital slide scanning system (Leica).

HALO software (Indica Labs) was used to perform single-cell analysis of the Vectra mIHC images. Cells were visualized based on nuclear and cytoplasmic stains, and mean pixel fluorescence intensity in the applicable compartments of each cell were measured (i.e., CD4 in the cytoplasmic compartment and FoxP3 in the nuclear compartment). A mean intensity threshold above background was used to determine positivity for each fluorochrome, thereby defining cells as either positive or negative for each marker. The data was then used to define co-localized populations, including PD-1^+^ CD4^+^ T cells, tumor cells, and Tregs. Spatial positions were extracted for each cell, and the spatial distances and ratios between these three cells types were calculated as detailed below. Performance of the *SpatialScore* biomarker was evaluated with the easyROC interface v1.3.1, available with the R package shiny (http://www.biosoft.hacettepe.edu.tr/easyROC/)^92^. CXCL13 IHC images were scored using a classifier method for the DAB stain based on optical density to obtain the positive percentage of the total mononuclear cell infiltrate per spot.

### CODEX antibodies

For CODEX, purified, carrier-free monoclonal and polyclonal anti-human antibodies were purchased from commercial vendors (Supplementary Table 2). The library of maleimide-modified short DNA oligonucleotides (TriLink) used for antibody conjugations was previously validated^18^. Conjugations were performed at a 2:1 weight/weight ratio of oligonucleotide to antibody, with at least 100 µg of antibody per reaction, as previously described^15,16,19^. All conjugated antibodies were validated and titrated under the supervision of a board-certified pathologist (C.M.S.), according to our recently published protocol^16^. Briefly, the conjugation of oligonucleotide to antibody was assessed using flow cytometry. Each conjugated antibody was then validated by staining an appropriate FFPE specimen with the antibody of interest, a positive control, and a negative control, using Alexa Flour 488, ATTO 550, and Alex Flour 647 as fluorescent reporters. Once validated, the staining for each conjugated antibody was optimized by starting at a dilution of 1:100 and titrating according to the signal-to-noise ratio. The specificity, sensitivity, and reproducibility of CODEX conjugated antibody staining has been demonstrated across multiple experiments in healthy and diseased tissues^15–19^.

### CODEX multiplex tissue staining and imaging

The CODEX experiment was performed, as previously described^15,16,19^. Briefly, the coverslip was deparaffinized and rehydrated, and heat-induced epitope retrieval was performed using Dako target retrieval solution, pH 9 (Agilent, #S236784-2) at 97 °C for 10 min. The coverslip was stained with an antibody cocktail with 54 antibodies (Supplementary Table 2) to a volume of 100 µl overnight at 4 °C in a sealed humidity chamber on a shaker. After multiple fixation steps using 1.6% paraformaldehyde, 100% methanol, and BS3 (Thermo Fisher Scientific, #21580), the coverslip was mounted onto a custom-made acrylic plate (Bayview Plastic Solutions). Imaging was performed with a Keyence BZ-X710 inverted fluorescence microscope equipped with a CFI Plan Apo λ 20x/0.75 objective (Nikon), an Akoya CODEX microfluidics instrument, and CODEX driver software v1.29.0.1 (Akoya Biosciences). Light exposure times and the arrangement of cycles are outlined in Supplementary Table 2. At the conclusion of the CODEX multicycle reaction, H&E staining was performed, and images were acquired in brightfield mode. Three experimental replicates were performed. As these experiments were run on consecutive tissue sections, there was minimal variation in cell density (number of cells/mm^2^; Supplementary Fig. 1d), cell composition, or cell localization between experiments. Experiment #1 had the highest mean density per tissue microarray spot and was selected for further processing and analysis.

### Data processing of CODEX images

Raw TIFF image files were processed using the CODEX Toolkit (github.com/nolanlab/CODEX), as previously described^15,16,19^. These images are hosted on ImmunoAtlas (https://immunoatlas.org/NOLN/210920-1/). After processing, the staining quality for each antibody was visually assessed in each tissue microarray spot, and cell segmentation was performed using the DRAQ5 nuclear stain. Marker expression was quantified, and single-cell data were saved as FCS files, which were then imported into CellEngine (cellengine.com) for cleanup gating. This resulted in a total of 117,170 cells across all tissue microarray spots.

After cleanup gating, FCS files were exported from CellEngine, imported into VorteX clustering software^93^, and subjected to unsupervised X-shift clustering using an angular distance algorithm. Clustering was based on all antibody markers except CD11b, CD16, CD164, CCR4, CCR6, EGFR, and p53. The optimal cluster number was guided by the elbow point validation tool in VorteX, resulting in 78 clusters. Clusters were manually verified and assigned to cell types based on morphology in H&E and fluorescent CODEX images and on their marker expression profiles. Clusters with similar features were merged, resulting in 21 cell type clusters. The expression frequencies of ICOS, IDO, Ki-67, and PD-1 were determined for the T cell and tumor cell clusters by manual gating in CellEngine for each tissue microarray spot, with visual comparison to the raw fluorescent image.

### Cellular neighborhood identification

CN identification was performed using a custom *k*-nearest neighbors’ algorithm in Python (github.com/nolanlab/NeighborhoodCoordination)^15^. For each of the 117,220 cells in this experiment, the window size was set at 10 (i.e., 1 center cell and its 9 nearest neighboring cells, as measured by the Euclidean distance between X/Y coordinates). We selected the 10-cell radius as a rough approximation of two cell distances from the center cell in each direction, which was visually determined to be a good indication of local functional activity. To identify 10 CNs, these windows were then clustered by the composition of their microenvironment with respect to the 21 identified cell types. This resulted in a vector for each window containing the frequency of each of the 21 cell types amongst the 10 neighborhoods. These windows were then clustered using Python’s *scikit-learn* implementation of MiniBatchKMeans with *k* = 10. Each cell was then allocated to the same CN as the window in which it was centered. In other words, CNs account for how the identity of the neighboring cell types impact the center cell’s function. All CN assignments were validated by overlaying them on the original fluorescent and H&E-stained images.

### Calculation of spatial distances and ratios between cell types

The X/Y coordinates for each cell type were determined during cellular segmentation, as described above. The minimal distance between each cell type and its nearest other cell types, and the averages of these minimal distances per tissue spot, were calculated in R (github.com/nolanlab/SpatialScore). Given our interest in the relationship of cell distances between three cell types (i.e., effector T cells (CT1), tumor cells (CT2) and Tregs (CT3)), we calculated the ratio of the minimal distances between CT1—CT2 (right distance) versus CT1—CT3 (left distance). This distance ratio represented the *SpatialScore*. To assess whether these ratios were significantly different from those of a random sample, we performed the following analysis per spot: For the number of CT1 cells in each spot, we randomly selected the same number of non-CT1 (nCT1) cells. For each of these nCT1 cells, we calculated the ratio of the minimal distances (nCT1—CT2 / nCT1—CT3) and determined the mean of this sample. We repeated this random sampling 100 times, and the average of all the means was reported. The distribution of the random values was assessed by the quant output variable, which indicates how many of the random means are smaller than the measured means. For instance, a quant of 97 indicates that 97% of the random means are smaller than the measured means. Thus, quant values closer to 100 or 0 indicate that the measured means are not random (Supplementary Fig. 4k-n).

### Sample size assessment for the *SpatialScore*

One hundred subsampling iterations of 50% and 75% of the pretreatment Vectra-derived *SpatialScore* data were performed. For each iteration, differences in the *SpatialScore* between responders and nonresponders were modeled using a linear mixed-effects model function [lmer(x ~response + (1 | patient), data = data.frame(x = SpatialScore_vector, response = compared_groups_vector, patient = patientID_vector))] taking a patient identifier as a random effect. The *p* values were derived using Satterthwaite’s degrees of freedom method, implemented in the lmerTest (v3.1.2)^94^ package. The *p* value of the statistical test in each iteration was plotted, and the paired mean *SpatialScore* in each iteration was visualized using the data analysis with bootstrap-coupled estimation (DABEST) package v0.3.0^67^. An exclusion analysis was also performed. Using the Vectra *SpatialScore* data, samples from each of the 14 patients were excluded over 14 iterations. The *SpatialScore* differences between responders and nonresponders were modeled using the linear mixed-effects models described above. The *p* value of the statistical test in each iteration was plotted, and the paired mean *SpatialScore* in each iteration was visualized using the data analysis with bootstrap-coupled estimation (DABEST) package v0.3.0^67^.

### Laser-capture microdissection (LCM)

Two serial sections of the tissue microarray were taken at 7 µm thickness and mounted onto frame slides with polyethylene naphthalate membranes (Thermo Fisher Scientific, #LCM0521). Slides were immersed for 20 s each in xylene (three times), 100% ethanol (three times), 95% ethanol (two times), 70% ethanol (two times), water, hematoxylin (Dako, #S3309), water, bluing reagent (Thermo Fisher Scientific, #7301), water, 70% ethanol (two times), 95% ethanol (two times), 100% ethanol (three times), and xylene (three times). Immediately after staining, cells were dissected from every tissue microarray spot on an ArcturusXT LCM System (Thermo Fisher Scientific) using the ultraviolet laser to cut out the desired region and the infrared laser to adhere the membrane to a CapSure HS LCM Cap (Thermo Fisher Scientific, #LCM0215). A tissue area containing roughly 1000 mononuclear cells was captured from each spot, with cell numbers determined based on density estimates by cell counting in an adjacent H&E-stained section. If a core had more than 1000 mononuclear cells, a tissue fragment containing around 1000 mononuclear cells was dissected from that core. If a core had less than 1000 cells, tissue fragments from corresponding cores on the serial section membrane were combined in the same LCM cap to obtain approximately 1000 cells. After microdissection, the caps were sealed using 0.5-ml tubes (Thermo Fisher Scientific, #N8010611) and stored at −80 °C until cDNA library preparation.

### Preparation of cDNA libraries and RNA sequencing

Sequencing libraries were prepared according to the Smart-3Seq protocol for LCM HS caps, as previously described with slight modifications^20^. Briefly, 10 µl of lysis mix consisting of 40% (v/v) 5 M trimethylglycine solution (Sigma, #B0300), 20% (v/v) 10 mM nuclease-free dNTP mix (Thermo Fisher Scientific, #R0192), 10% (v/v) 20 µM first-strand primer in TE buffer (1 S, /5Biosg/GT GAC TGG AGT TCA GAC GTG TGC TCT TCC GAT CTT TTT TTT TTT TTT TTT TTT TTT TTT TTT TV; Integrated DNA Technologies), 10% (v/v) Triton-X 100 (Sigma #T8787; diluted to 0.5% v/v in molecular biology-grade water), and 20% (v/v) Proteinase K (New England Biolabs, #P8107S; diluted to 0.125 mg/ml in in molecular biology-grade water) was added to the center of each LCM cap. Caps were sealed with 0.2 ml low-retention PCR tubes (Corning, #PCR-02-L-C) and incubated on a pre-warmed metal CapSure incubation block (Thermo Fisher Scientific, #LCM0505) at 60 °C in an incubator. Then, tubes were briefly centrifuged, and 10 µl of template-switching reverse-transcription (TS-RT) FFPE LCM mix consisting of 40% (v/v) 5x SMARTScribe first-strand reaction buffer (Clontech, #639537), 20% (v/v) 20 mM DTT (Clontech, #639537), 10% (v/v) 20x RNase inhibitor (Thermo Fisher Scientific, #AM2694), 4% (v/v) 50 µM second-strand primer in TE buffer (2 S, /5Biosg/CT ACA CGA CGC TCT TCC GAT CTN NNN NrGrG rG; Integrated DNA Technologies), 4% (v/v) 200 mM MgCl_2_ (Sigma, #63069), 2% 5 mM proteinase K inhibitor (EMD Millipore, #539470), and 20% (v/v) 100 U/µl SMARTScribe reverse transcriptase (Clontech, #639537) was added. Samples were incubated in a programmable thermal cycler (42 °C for 30 min, 70 °C for 10 min, 4 °C hold), and 1.25 µl of a unique P5 primer and 1.25 µl of a universal P7 primer (2 µM in TE buffer each; Integrated DNA Technologies; sequences available upon request) and HiFi HotStart ReadyMix (Kapa, #KK2601) were then added, followed by 22 cycles of PCR amplification (98 °C for 45 s; 22 cycles at 98 °C for 15 s, 60 °C for 30 s, 72 °C for 10 s; then 72 °C for 60 s, and 4 °C hold). Amplified cDNA was next purified with SPRI bead mix (Beckman Coulter, #B23317) and a magnetic separation block (V&P Scientific, #VP772F4). Finally, the samples were washed with 80% ethanol and resuspended in TE buffer to yield the sequencing-ready library.

Libraries were profiled for size distribution on an Agilent 2200 TapeStation with High Sensitivity D1000 reagent kits and quantified by qPCR with a dual-labeled probe as previously described^95^. Libraries were excluded if <40% of their transcripts were within a 165-500 bp range. A total of 64 libraries were mixed to equimolarity, according to the qPCR measurements. The RNA libraries were sequenced on an Illumina NextSeq 500 instrument with a High Output v2.5 reagent kit (Illumina, #20024906) to a minimum sequencing depth of 1.5 M reads per sample (mean: 3.7 M) and minimum uniquely aligned reads of 364,468 per sample (mean: 916,607) using read lengths of 76 nucleotides (nt) for read 1 and 8 nt for read 2. On average, we obtained reads from 11,166 genes per sample (median: 11,267) and 379,615 unique transcripts per sample (median: 336,005), which is comparable to previously published FFPE-based RNA-seq studies of human cancers^20^.

### Processing of RNA-seq data

Base calls from the NextSeq were de-multiplexed and converted to FASTQ format with bcl2fastq (NextSeq system suite software, Illumina v2.20.0.422). The five-base unique molecular identifier (UMI) sequence and the G-overhang were extracted from FASTQ data, and A-tails were removed with umi_homopolymer.py (github.com/jwfoley/3SEQtools). Reads were aligned and further processed to remove duplicates using STAR v2.7.3a (github.com/alexdobin/STAR)^96^. Bulk gene expression profiles were transcript per million (TPM) normalized and log2 transformed. Differences in *CXCL13* and *CXCR5* expression between groups were modeled with Linear Mixed Effects Models on a per spot basis using the lmer function from package lme4 (v1.1.21)^97^ and taking the patient intercept as a random effect. The pairwise *p* values were derived from t-ratio statistics in the contrast analysis using the lmerTest (v3.1.2)^94^ and corrected for multiple hypothesis testing using the Holm Bonferroni method implemented in the model-based (v0.1.2) package (github.com/easystats/modelbased).

### Principal component analysis (PCA) immune scores

PCA scores and principal component 1 (PC1) coefficients were computed for the normalized bulk RNA-seq data on a per spot basis using the prcomp function in base R. The IFN-γ score was calculated using the six gene signature published by Ayers et al. ^39^. The TGF-β score was calculated using the 15 gene signature published by Mariathasan et al. ^40^. The immune activation and immunosuppression scores were computed using the genes listed in Supplementary Table 3c-d. Differences in PC1 scores between patient groups were modeled using Linear Mixed Effects Models on a per spot basis using the lmer function [lmer(x ~pre + (1 | patient), data = data.frame(x = PCA_scores_vector, pre = compared_groups_vector, patient = patientID_vector))] from package lme4 (v1.1.21)^97^ and taking the patient intercept as a random effect. Differences between responders and nonresponders, as well as pretreatment and post-treatment samples were modeled as fixed effects and tested using Satterthwaite’s degrees of freedom method. The pairwise *p* values were derived from t-ratio statistics in the contrast analysis using the lmerTest (v3.1.2)^94^ and corrected for multiple hypothesis testing using the Holm Bonferroni method implemented in the modelbased (v0.1.2) package (github.com/easystats/modelbased).

### Identifying bulk RNAseq gene signatures associated with tumor cells and the *SpatialScore*

LASSO regression models were used to find genes predictive of tumor cells and the *SpatialScore*. These models were estimated using the LassoCV object in the scikit-learn python package v0.22. Six-fold cross validation was used to select the optimal regularization parameter. Specifically, an L1-regularized linear model was fit to predict the frequency of tumor cells from the gene expression data per tissue microarray spot. For this model, the response variable was the log-transformed per spot percentage of tumor cells. A pseudo-count of 1% was added to genes. The features utilized as predictors were the per spot log-transformed TPM counts and the log frequency of CD4^+^ T cells. Genes with positive nonzero coefficients were interpreted as positively associated with tumor cells. Similarly, an L1-regularized linear model was fit to predict the *SpatialScore* from the gene expression data on a per spot basis. For this model, the response variable was the log-transformed *SpatialScore* distance ratio. The features used as predictors were the per spot log-transformed TPM counts. Genes with nonzero coefficients were selected in figures as predictive.

For both models, six cross-validation folds were used to determine the regularization parameter. For the model predicting tumor cell frequency, the selected regularization parameter was 0.028 and the average validation set mean squared error (MSE) across folds was 0.31. For the model predicting distance ratio, the selected regularization parameter was 0.300 and the average validation set MSE across folds was 0.71.

### CIBERSORTx signature matrix

To generate a CSx signature matrix, we used a publicly available scRNA-seq dataset from Gaydosik et al. ^62^ that was obtained from skin biopsies of five CTCL patients. Datasets were downloaded from the Gene Expression Omnibus (GEO) database (accession code GSE128531), and single-cell profiles were combined and analyzed using the Seurat R package (v3.1.4)^98^. Cells with between 500 and 7500 genes detected and less than 10% mitochondrial transcripts were included in the analysis.

Data were log10normalized and clustered with the Louvain method^99^ based on the first 13 PCs and resolution of 1.8. Cell clusters were visualized using Uniform Manifold Approximation and Projection (UMAP)^100^, with the same PCs. Major cell types were assigned according to the expression of corresponding marker genes (Supplementary Fig. 6a, Supplementary Table 4a). Fibroblasts and pericytes were merged into a stromal cluster. A full list of differentially expressed genes between the 10 annotated clusters is available in Supplementary Data 7. The T cell cluster was divided into CD4^+^ T cells, CD8^+^ T cells, Tregs, γδ T cells, and tumor cells based on the expression of certain T cell and tumor marker genes (Supplementary Fig. 6b, Supplementary Fig. 7, Supplementary Table 4b). Tumor cells from patient CTCL-5 were excluded due to extreme heterogeneity; these tumor cells were characterized by high expression of NK cell markers (e.g., *NKG7*, *GNLY*), suggesting NK T cell lineage T cell clustering was based on the first 15 PCs and resolution of 1.9. The same PCs were used to generate UMAP projections for the T cell clusters. A matrix of single cells and their assigned cell type identities were used to create a signature matrix (Supplementary Data 8) using CSx (v.1.0) with fractions mode on the CSx website (arguments used: single_cell = TRUE; code available from cibersortx.stanford.edu/)^21^. There was a good correlation between the CSx and CODEX cell type clusters (Fig. 5h, Supplementary Fig. 8).

### CIBERSORTx deconvolution

The signature matrix was used to deconvolve tumor cell gene expression in CSx (v.1.0) with the HiRes deconvolution mode using CSx website (arguments used: rmbatchSmode = T, QN = F)^21^. Log2 fold changes were computed for every deconvolved gene across patient groups. Differences in gene expression between patient groups were modeled with Linear Mixed-Effects Models on a per spot basis using the lmer function [lmer(x ~ pre + (1 | patient), data = data.frame(x = gene_expression_vector, pre = compared_groups_vector, patient = patientID_vector))] from package lme4 (v1.1.21)^97^ and taking the patient intercept as a random effect. The *p* values were derived using Satterthwaite’s degrees of freedom method, implemented in the lmerTest (v3.1.2)^94^ package. The *p* values were adjusted with the Benjamini-Hochberg correction using the p.adjust function in R. Volcano plots were generated using the ggplot2 (v3.3.0)^101^ and ggrepel (v0.8.1)^102^ packages in R. Genes with Benjamini-Hochberg-adjusted *p* < 0.1 were considered significant (Fig. 5i–j, Supplementary Fig. 6d).

CSx-deconvolved *CXCL13* expression in tumor cells was log2 transformed on a per spot basis. Differences in *CXCL13* expression between patient groups were modeled using Linear Mixed-Effects Models on a per spot basis using the lmer function [lmer(CXCL13 ~ pre + (1 | patient), data = data.frame(CXCL13 = CXCL13_expression_vector, pre = compared_groups_vector, patient = patientID_vector))] from package lme4 (v1.1.21) and taking the patient intercept as a random effect. The pairwise *p* values were derived from t-ratio statistics in the contrast analysis using the lmerTest (v3.1.2) and corrected for multiple hypothesis testing using the Holm Bonferroni method implemented in the model-based (v0.1.2) package (github.com/easystats/modelbased). To examine *CXCL13* expression in tumor cells on a per patient basis, the mean from biological replicates was computed before plotting the log2 normalized CSx-deconvolved *CXCL13* expression. The Wilcoxon signed-rank test was used to evaluate whether patient-matched CSx-deconvolved CXCL13 expression in the tumor cells was different between groups.

### Statistics and reproducibility

Statistical analyses were performed with R and Prism v8 (GraphPad Software, Inc). Results with *p* < 0.05 were considered significant, unless otherwise stated. The significance of pretreatment differences between individual responder and nonresponder patients was tested using a two-sided Wilcoxon’s rank-sum test. Pre- to post-treatment pairwise statistical significance for individual patients was tested using a two-sided Wilcoxon’s signed-rank test. For differences across patient groups (i.e., responders versus nonresponders pretreatment, responders pre- versus post-treatment, nonresponders pre- versus post-treatment, and responders versus nonresponders post-treatment), the significance was tested using a linear mixed-effect model with Holm Bonferroni multiple hypothesis testing corrections. For *SpatialScore* comparisons between responders and nonresponders pretreatment (comparison 1) or post-treatment (comparison 2), the significance was tested using a linear mixed-effect model taking a patient identifier as a random effect. Multiple hypothesis testing was not employed for this analysis since the two comparisons were independent: the pretreatment comparison assessed the ability of the *SpatialScore* to predict therapeutic response, whereas the post-treatment comparison assessed the influence of therapy on the *SpatialScore*. For subsampling and exclusion analyses of the pretreatment Vectra-derived *SpatialScore* differences between responders and nonresponders, the significance was tested using a linear mixed-effect model taking a patient identifier as a random effect. For differential gene expression between patient groups in CSx deconvoluted expression profiles, the significance was tested using linear mixed-effect models, where *p* values were adjusting using Benjamini-Hochberg correction and results with *p* < 0.1 were considered significant. Correlations were evaluated with the non-parametric Spearman test. The investigators were not blinded to allocation during experiments and outcome assessment. No sample-size estimates were performed to ensure adequate power to detect a pre-specific effect size. *p < 0.05, **p < 0.01, ***p < 0.001, ****p < 0.0001.

For CODEX, three experimental replicates were performed on consecutive tissue sections. As shown in Supplementary Fig. 1d, experiment #1 had the highest mean cell density per tissue microarray spot and was therefore selected for further processing and analysis. The *SpatialScore* biomarker that was identified with CODEX was reproduced and validated using the Vectra mIHC platform in one experiment. Further reproducibility studies were not conducted.

### Reporting Summary

Further information on research design is available in the Nature Research Reporting Summary linked to this article.

## Supplementary information

Supplementary Information

Description of Additional Supplementary Files

Supplementary Data 1

Supplementary Data 2

Supplementary Data 3

Supplementary Data 4

Supplementary Data 5

Supplementary Data 6

Supplementary Data 7

Supplementary Data 8

Reporting Summary

## Data Availability

CODEX imaging data generated in this study have been deposited in the ImmunoAtlas public repository [https://immunoatlas.org/NOLN/210920-1]. RNA-seq data generated in this study have been deposited in the GEO database under accession code GSE162137. The CTCL scRNA-seq publicly available data used in this study are available in the GEO database under accession code GSE128531. Source data are provided with this manuscript. The remaining data are available within the Article, Supplementary Information or Source Data file. Source data are provided with this paper.

## Source data

Source Data

## Code availability

SpatialScore code: https://github.com/nolanlab/SpatialScore. All other code used in this study is previously published and specified in the Methods section.
