# Supplementary material for: Immune cell topography predicts response to PD-1 blockade in cutaneous T cell lymphoma
Source: Nat Commun. 2021 Nov 18;12:6726. doi: 10.1038/s41467-021-26974-6 (PMC8602403; doi:10.1038/s41467-021-26974-6)
Supplement: Supplementary file 1 — Supplementary Information [file 41467_2021_26974_MOESM1_ESM.pdf]

Immune cell topography predicts response to PD-1 blockade in cutaneous T cell lymphoma

SUPPLEMENTARY INFORMATION

Supplementary Figs. 1-8 and Supplementary Tables 1a-b, 2, 3a-f, and 4a-b.

Supplementary Figure 1

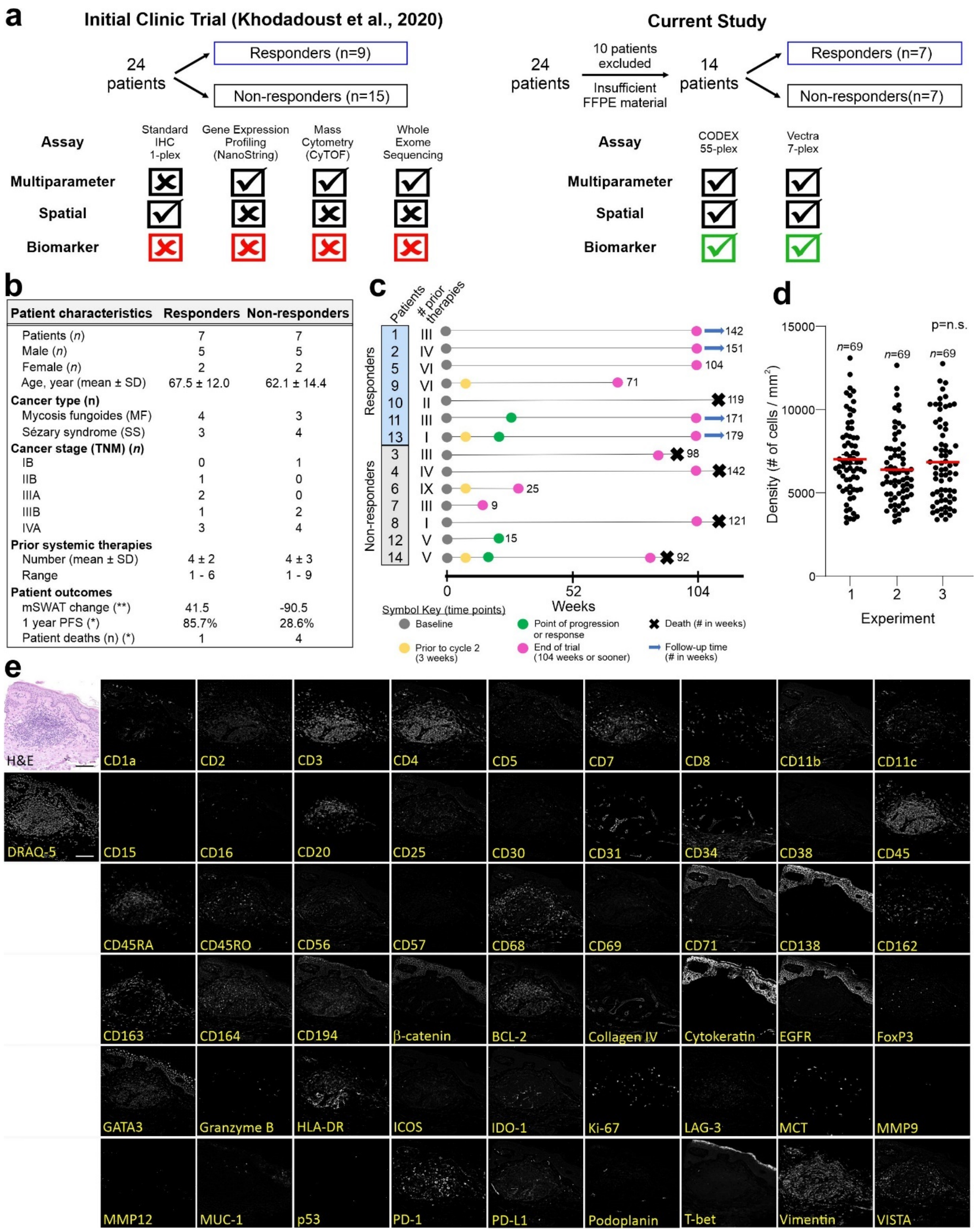

Supplementary Figure 1. Study design and CODEX experimental validation. **a**, Patient cohorts and comparison of biomarker assay performance in the initial pembrolizumab clinical trial (*n*=24 CTCL patients)[1] and the current study (*n*=14 CTCL patients). **b**, Patient characteristics. Significant differences between responders (*n*=7) and non-responders (*n*=7) were only observed for patient outcomes: change from baseline in skin by the modified Severity Weighted Assessment Tool (mSWAT) (**, *p*=0.0019 by two-sided Wilcoxon's rank sum test), 1 year progression free survival (PFS) (*, *p*=0.0306 by Wilcoxon's rank sum test), and patients deaths (*, *p*=0.0168 by log rank test in Kaplan-Meier overall survival curve; see **Fig. 1b**). **c**, Swimmer plot for individual patients, depicting treatment history and timepoint for every tumor biopsy used in this study (lines represent overall survival in weeks; a rightward arrow indicates that the patient was alive following the final tumor biopsy; a rightward X indicates patient death). **d**, Cell densities (number of cells/mm^2) per tissue microarray spot over three CODEX experiments. The mean density (red bar) was 7021.0 cells/mm^2 for experiment #1, 6388.9 cells/mm^2 for experiment #2, and 6849.4 cells/mm^2 for experiment #3; computational analyses were performed on data acquired in experiment #1. *P* value calculated with a two-sided Friedman test (*p*=n.s.). **e**, Validation of the 56-marker CODEX antibody panel used to stain the FFPE CTCL skin tissue microarray. H&E staining and false gray images for each marker are shown for a single tissue microarray spot. Scale bars, 100 µm. Source data are provided as a Source Data file.

Supplementary Figure 2

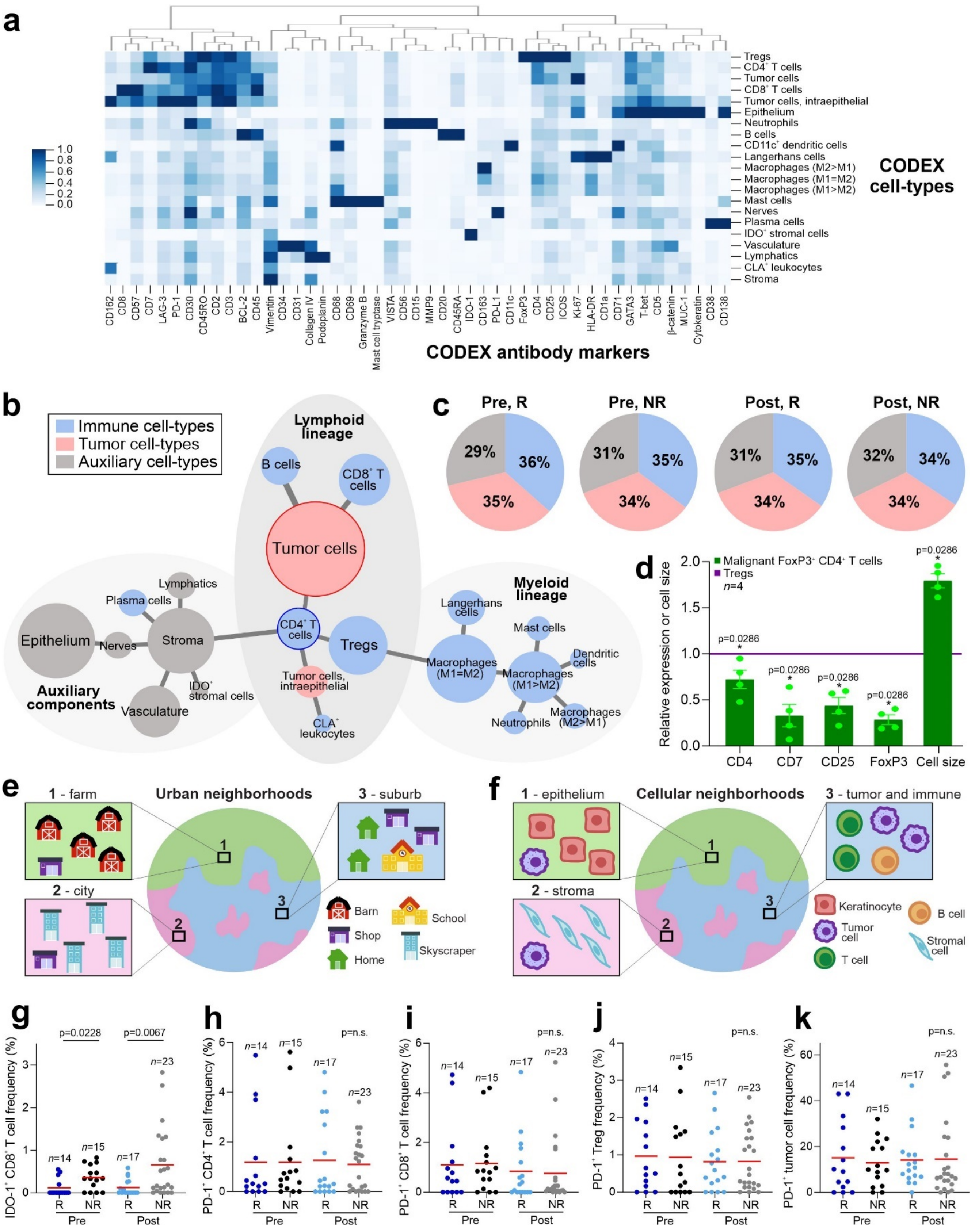

Supplementary 2. Characterizing CODEX cell-types, the cellular neighborhood concept, and checkpoint frequencies. **a**, Heatmap of CODEX antibody marker expression in each of the 21 identified cell-types. **b**, Minimal spanning tree of the 21 cell-types, which are colored blue (immune cell-types), red (tumor cell-types) and gray (auxiliary cell-types). **c**, Pie charts of the frequencies of tumor, immune and auxiliary cell-types for each patient group (p=n.s. for all comparisons). **d**, Mean expression of select markers on malignant FoxP3$^+$ CD4$^+$ T cells (green bars, mean ± s.e.m.) relative to Tregs (purple line) per tissue microarray spot (light green circles) in patient 2; mean fold-changes are 0.72 for CD4, 0.38 for CD7, 0.44 for CD25, 0.29 for FoxP3, and 1.79 for cell size. *P* values calculated by two-sided Wilcoxon's rank-sum tests. **e-f**, Conceptual neighborhood schematic showing that urban neighborhoods are determined based on their composition of buildings (**e**) and cellular neighborhoods based on their composition of cell-types (**f**). **g-k**, Frequencies of IDO-1$^+$ CD8$^+$ T cells (**g**), PD-1$^+$ CD4$^+$ T cells (**h**), PD-1$^+$ CD8$^+$ T cells (**i**), PD-1$^+$ Tregs (**j**), and PD-1$^+$ tumor cells (**k**) per tissue microarray spot across patient groups (mean, red bar). *P* values calculated with a linear mixed-effect model with Bonferroni's corrections for multiple comparisons. Source data are provided as a Source Data file.

Supplementary Figure 3

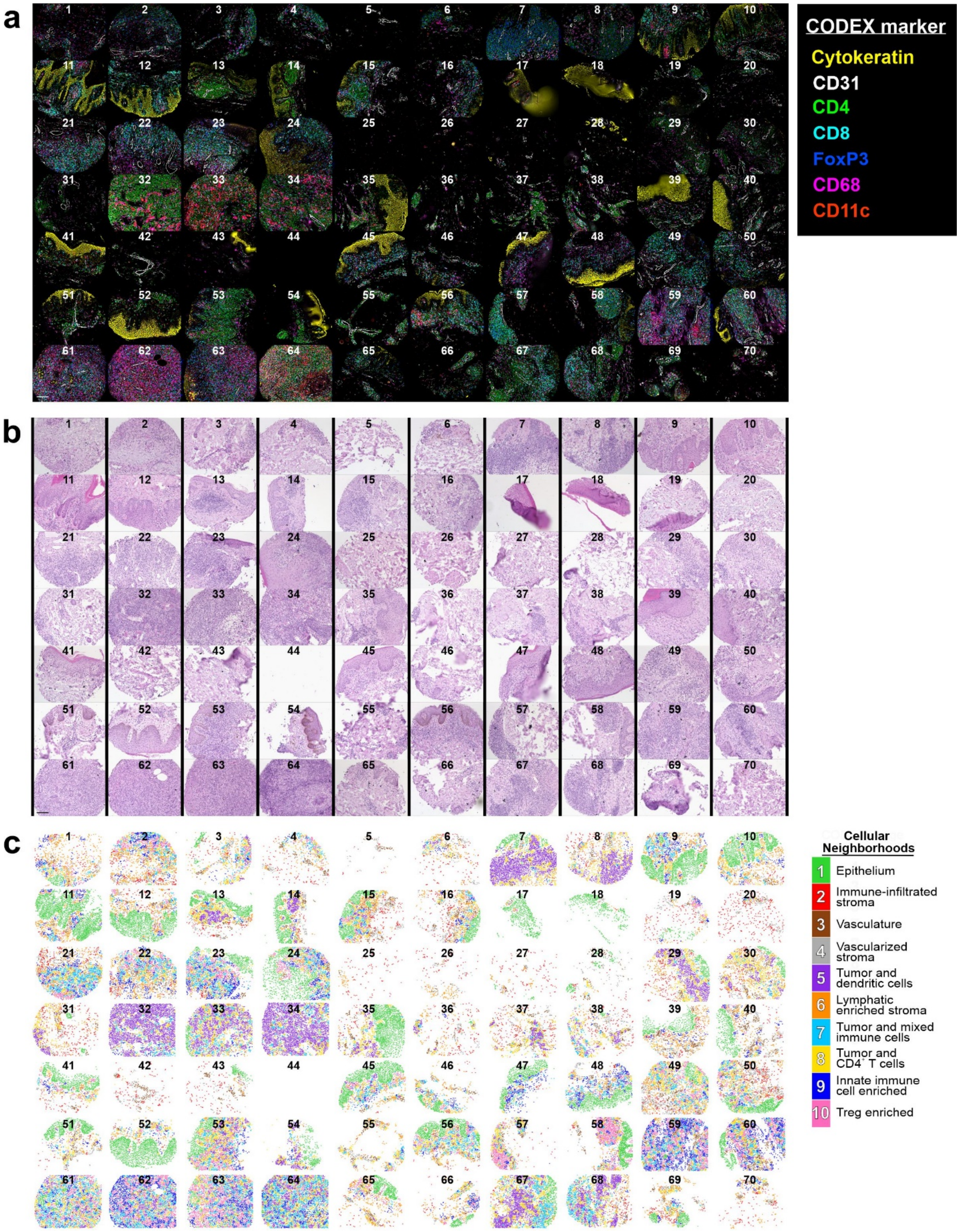

Supplementary Figure 3. CODEX, H&E, and cellular neighborhood images for the CTCL tissue microarray. **a**, CODEX seven-color overlay image of the FFPE CTCL tissue microarray. Scale bars, 100 µm. **b**, Corresponding H&E images of the tissue microarray. Scale bars, 100 µm. **c**, Dot plot Voronoi diagrams of the 10 identified cellular neighborhoods (CNs), colored according to the corresponding legend. Note, tissue microarray spot 44 was lost during sectioning.

Supplementary Figure 4

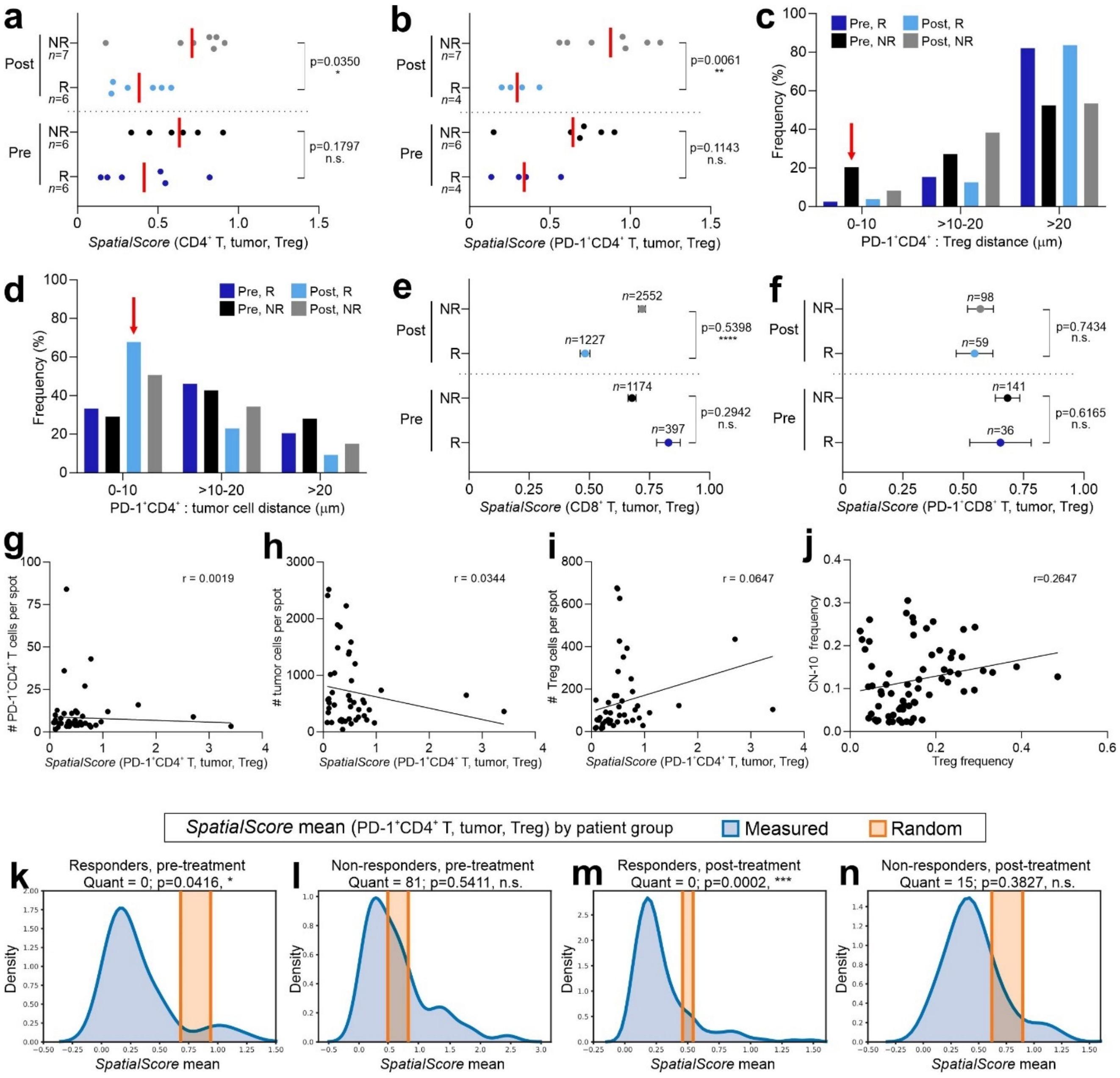

Supplementary Figure 4. Detailing the *SpatialScore* in CTCL. a-b, *SpatialScore* calculated per patient for all $CD4^+$ T cells (**a**) and PD-1^+ $CD4^+$ T cells (**b**) (mean, red bar). *P* values calculated by two-sided Wilcoxon's rank-sum tests, with no adjustments for multiple hypotheses. **c**, Frequency distribution of the physical distances in the tissue between PD-1^+ $CD4^+$ and Tregs by patient group, with the red arrow showing that these two cell-types are closest in non-responders pre-treatment. **d**, Frequency distribution of the distances between PD-1^+ $CD4^+$ and tumor cells by patient group, with the red arrow showing that these two cell-types are closest in responders post-treatment. **e-f**, *SpatialScore* calculated per cell for all $CD8^+$ T cells (**e**) and PD-1^+ $CD8^+$ T cells (**f**) (mean ± s.e.m.). *P* values calculated with a linear mixed-effect model taking a patient identifier as a random effect. **g-i**, Correlations of the number of PD-1^+ $CD4^+$ T cells (**g**) tumor cells (**h**), and Tregs (**i**) relative to the *SpatialScore* per tissue microarray spot. Correlations evaluated with a two-sided Spearman test. **j**, Correlation of the frequency of CN-10 (Treg enriched neighborhood) relative to the frequency of Tregs as a function of all immune cells. Correlations was evaluated with the Spearman test. **k-n**, Density plots of the measured mean *SpatialScore* distribution (blue) and corresponding random mean with its standard deviation (orange) for responders pre-treatment (**k**) non-responders pre-treatment (**l**) responders post-treatment (**m**), and non-responders post-treatment (**n**). *P* values calculated by two-sided Wilcoxon's rank-sum tests. The Quant values correspond to the percentage of randomly measured values that are smaller than the measured value; values closer to 0 or 100 indicate that the measurement is not random. Source data are provided as a Source Data file.

Supplementary Figure 5

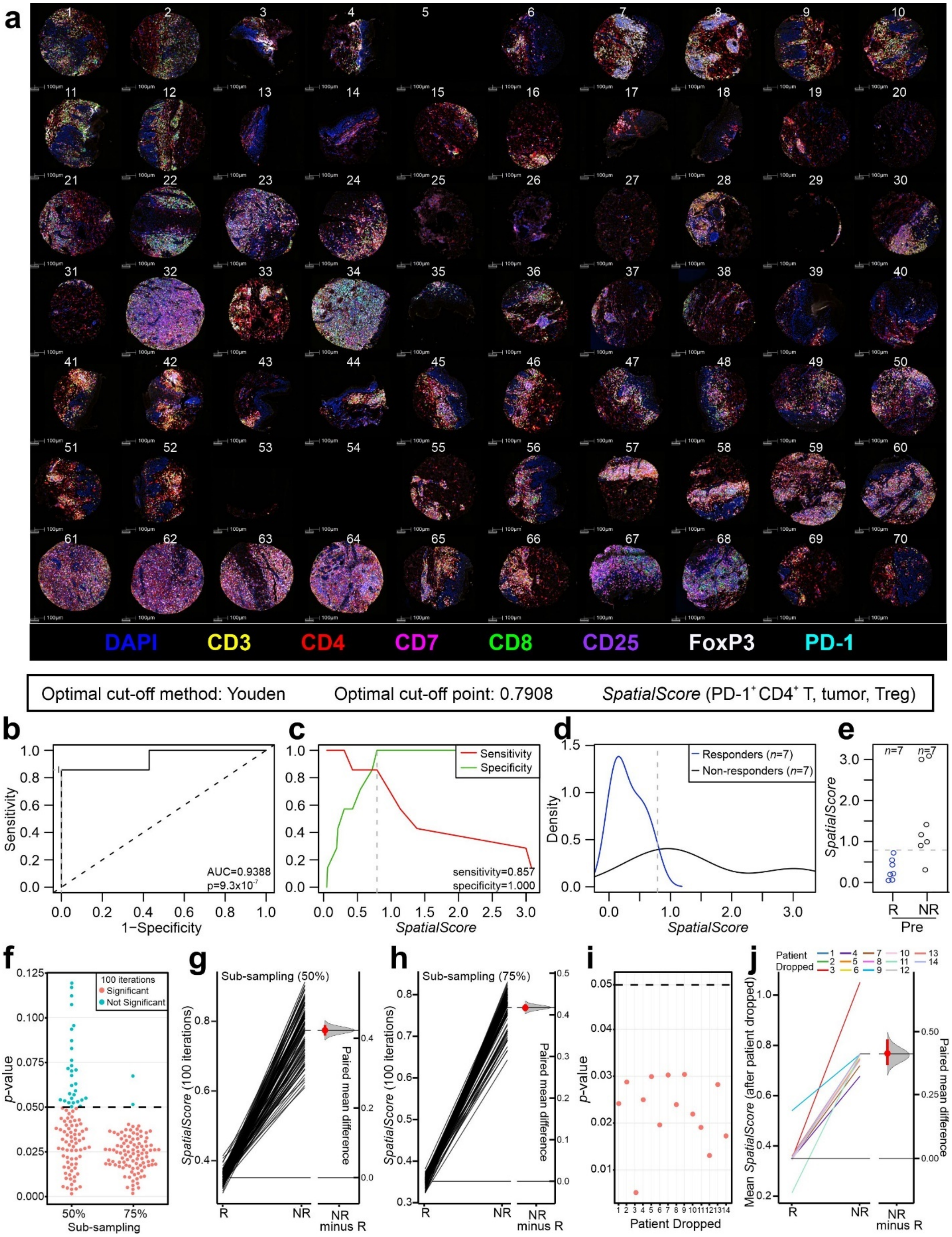

Supplementary Figure 5. Validation of the *SpatialScore* using a clinically accessible mIHC platform. a, Vectra mIHC staining of the CTCL tissue microarray with an eight-color overlay image, including DAPI (blue), CD3 (yellow), CD4 (red), CD7 (magenta), CD8 (green), CD25 (purple), FoxP3 (white), and PD-1 (cyan). Scale bars, 100 µm. **b-e**, Biomarker performance measures, using an optimal cut-off point of 0.7908, for the pre-treatment *SpatialScore* calculated from data obtained with the Vectra mIHC platform including a receiver operating characteristics (ROC)curve (**b**), sensitivity and specificity plots (**c**), density plots (**d**), and patient-based scatter plots (**e**), whereby 100% of responders were below the *SpatialScore* cut-off point and 85.7% of non-responders were above the cut-off point. **f**, Scatter plots of *p* values across 100 iterations of random subsamples of 50% and 75% of the pre-treatment Vectra-derived *SpatialScore* data between responders and non-responders. Differences between patient groups were modeled with a linear mixed-effects model taking a patient identifier as a random effect and *p* values were derived using Satterthwaite's degrees of freedom method. **g-h**, Paired plots of the mean *SpatialScore* between responders and non-responders across 100 iterations of subsampling of 50% (**g**) and 75% (**h**) for the full dataset (left) and distributions of the paired mean *SpatialScore* differences (right). The mean difference (i.e., effect size) is indicated with a red circle and the bootstrap 95% confidence interval is illustrated by the red vertical line. **i**, Dot plot of *p* values for the *SpatialScore* between responders and non-responders calculated from the Vectra dataset across 14 iterations of individual patient exclusion. Differences between patient groups were modeled with a linear mixed-effects model taking a patient identifier as a random effect and *p* values were derived using Satterthwaite's degrees of freedom method. **j**, Paired plot of the mean *SpatialScore* between responders and non-responders, where each colored line corresponds to the *SpatialScore* after that patient was excluded (left), and distribution of the paired mean *SpatialScore* differences (right). The mean difference (i.e., effect size) is indicated with a red circle and the bootstrap 95% confidence interval is illustrated by the red vertical line. Source data are provided as a Source Data file.

Supplementary Figure 6

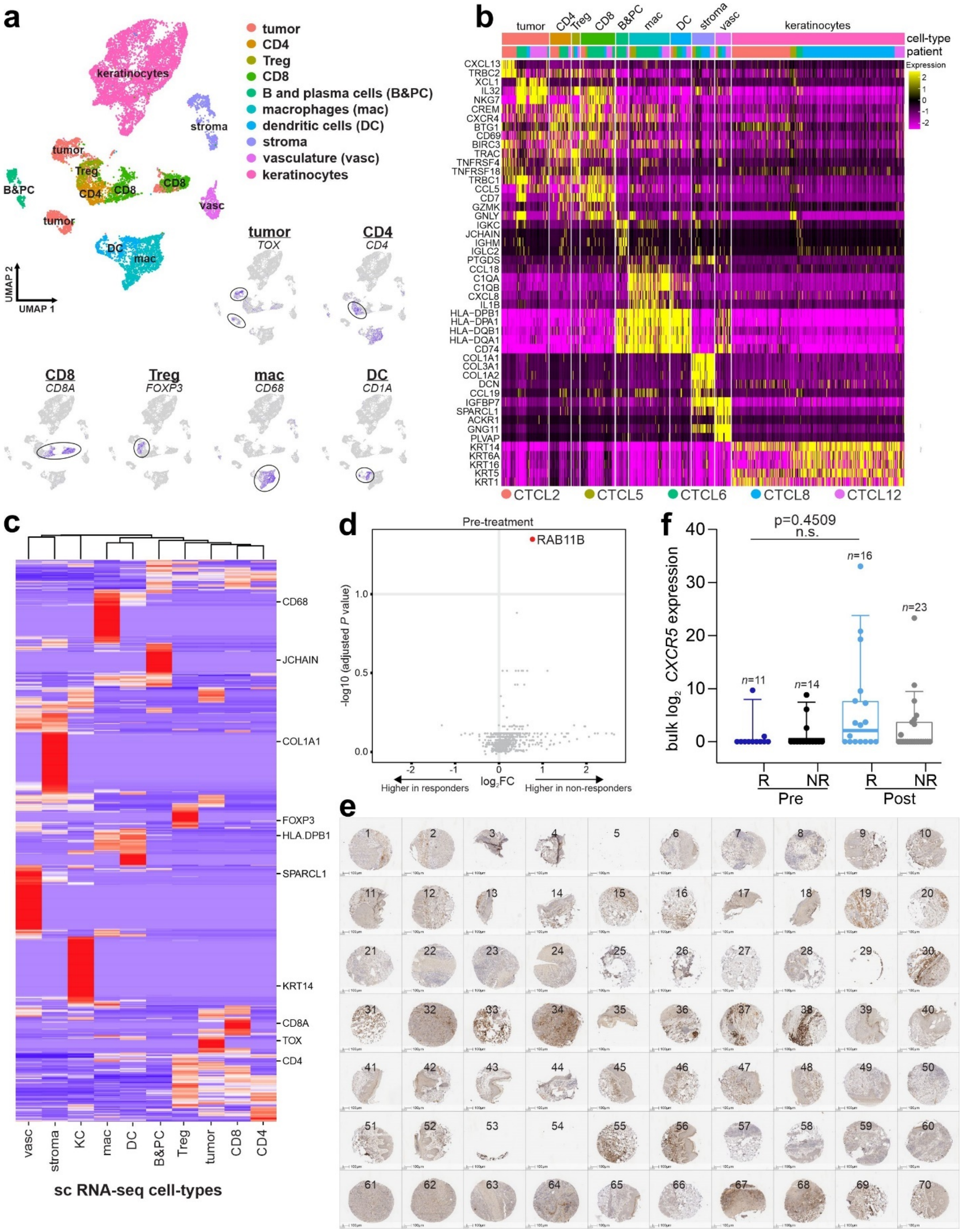

Supplementary Figure 6. Characterizing CIBERSORTx cell-types and CXCL13 expression patterns. a, UMAP of 10 major cell-types identified in the CTCL scRNA-seq dataset from Gaydosik et al.[2],with feature plots showing expression for a subset of marker genes including *TOX* (tumor cells), *CD4* ($CD4^+$ T cells), *CD8A* ($CD8^+$ T cells), *FOXP3* (Tregs), *CD68* (macrophages), and *CD1A* (dendritic cells). **b**, Heatmap of the top differentially expressed genes (rows) for the 10 cell-types (top color bar) and by patient from the scRNA-seq dataset[2] (second color bar, legend below heatmap). **c**, Signature matrix, highlighting key marker selected genes (rows), used to enumerate cell-type fractions and resolve gene expression profiles from CTCL bulk RNA-seq (also see **Supplementary Data 8**). **d**, Differential expression of CSx-resolved tumor cell genes in responders versus non-responders pre-treatment. *P* values calculated with a linear mixed-effect model with Benjamini-Hochberg correction (significantly different genes ($p < 0.1$) are colored red). **e**, CXCL13 IHC staining of the tissue microarray. Scale bars, 100 µm. **f**, Normalized bulk *CXCR5* gene expression per tissue microarray spot across patient groups. Boxes, median ± upper and lower quartiles; whiskers, 1.5x IQR. *P* values were calculated with a linear mixed-effect model with Bonferroni's corrections for multiple comparisons. Source data are provided as a Source Data file.

Supplementary Figure 7

a

Cell type (cluster #)	Reactive TIL markers	CD7 level	Tumor, proliferation, cell cycle progression, and survival markers
**CD4 T cells # 2, 15**	CD4^{hi}, CD69^{hi}, STAT4hi	low	low
**CD8 T cells # 4, 11, 12**	CD8A^{hi}, CD8B^{hi}	hi	low
**Treg cells # 17**	CD4^{hi}, FOXP3^{hi}, ICOS^{hi}, IL2RA^{hi}	low	low
**γδ T cells # 9, 19, 24**	TRDC^{hi}	variable	low
**Tumor cells # 6, 14, 18, 20, 22, 23, 26**	variable	low	high
**CTCL-5 tumor cells # 0, 1, 3, 5, 7, 8, 10, 13, 16, 25**	NKG7^{hi}, GNLY^{hi}	hi	high
**Unclassified # 21**	CD8A^{hi}, CD8B^{hi}	low	high

b

c

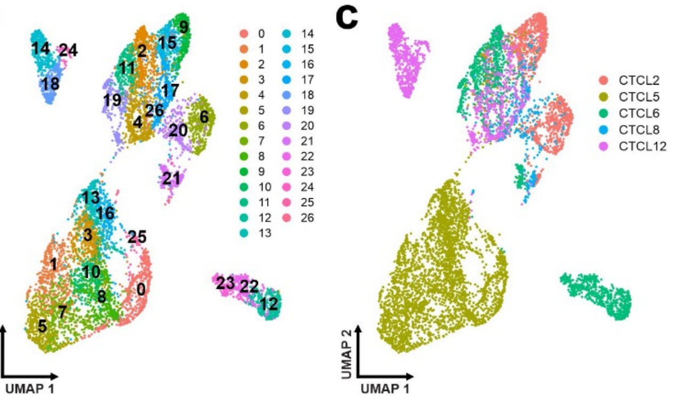

d

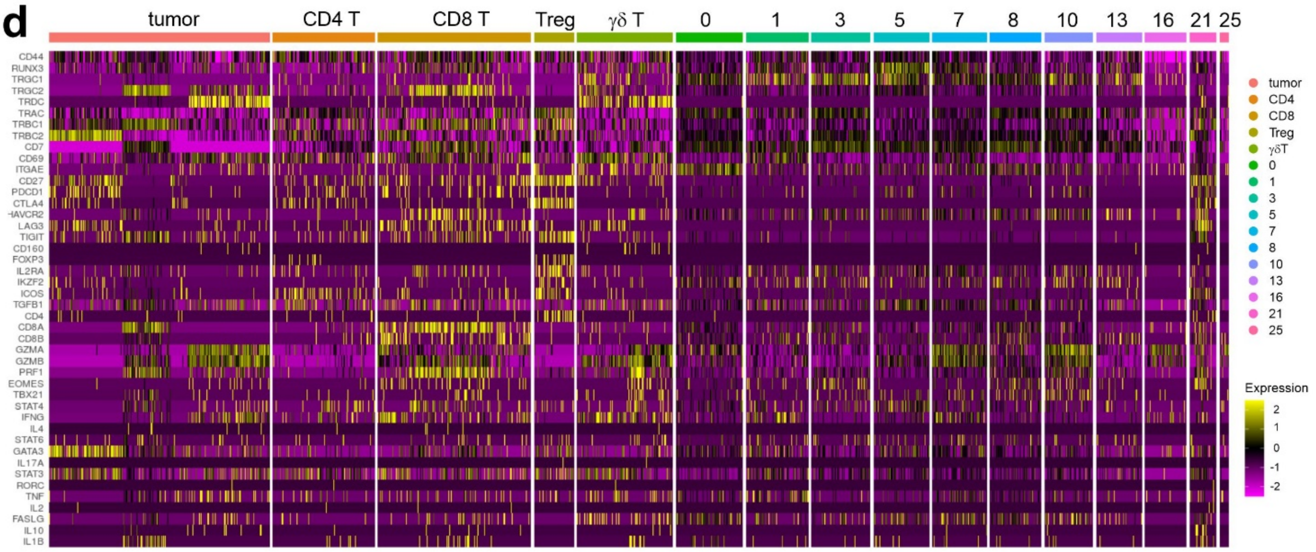

e

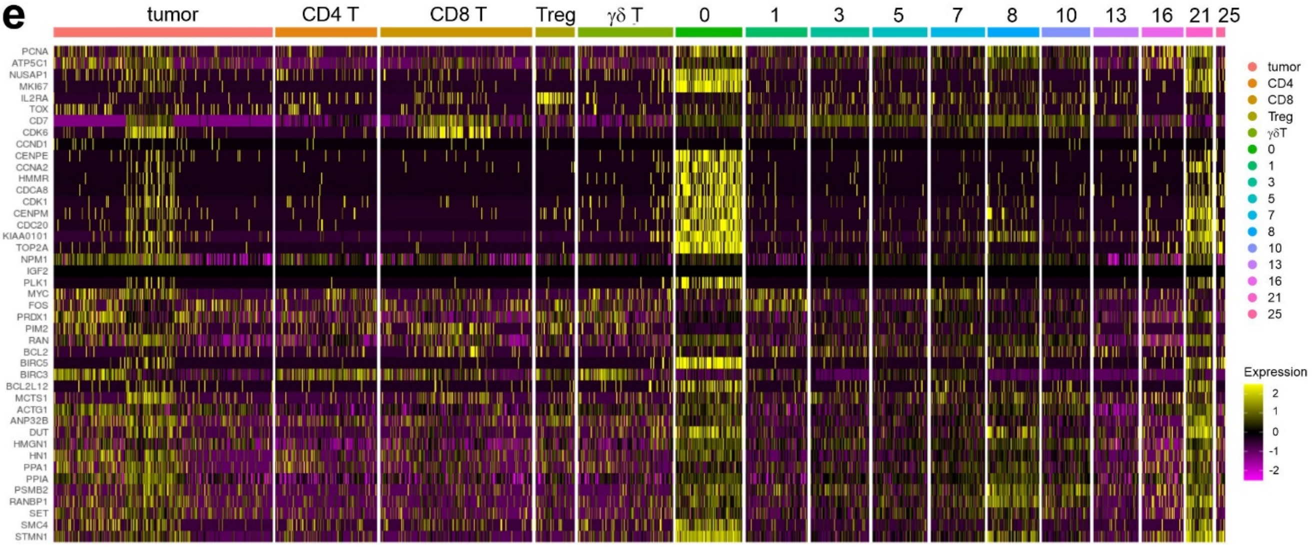

Supplementary Figure 7. Distinguishing reactive tumor infiltrating lymphocytes from tumor cells in scRNA-seq data. Clustering and cluster annotation were performed on the CTCL scRNA-seq dataset from Gaydosik et al.[2]. **a**, Table of 27 annotated T cell clusters, which summarizes the marker differential expression patterns between tumor infiltrating lymphocytes and tumor cells. **b**, UMAP embedding of 27 T cell clusters detected using Louvain clustering. **c**, UMAP embedding colored for each of the 5 patients included in the published scRNA-seq dataset[2]; our analysis excluded tumor cells from patient CTCL-5 due to extreme heterogeneity. **d**, Heatmap showing T cell marker gene expression by cluster. **e**, Heatmap showing tumor, proliferation, cell cycle progression, and survival marker expression by cluster. Source data are provided as a Source Data file.

Supplementary Figure 8

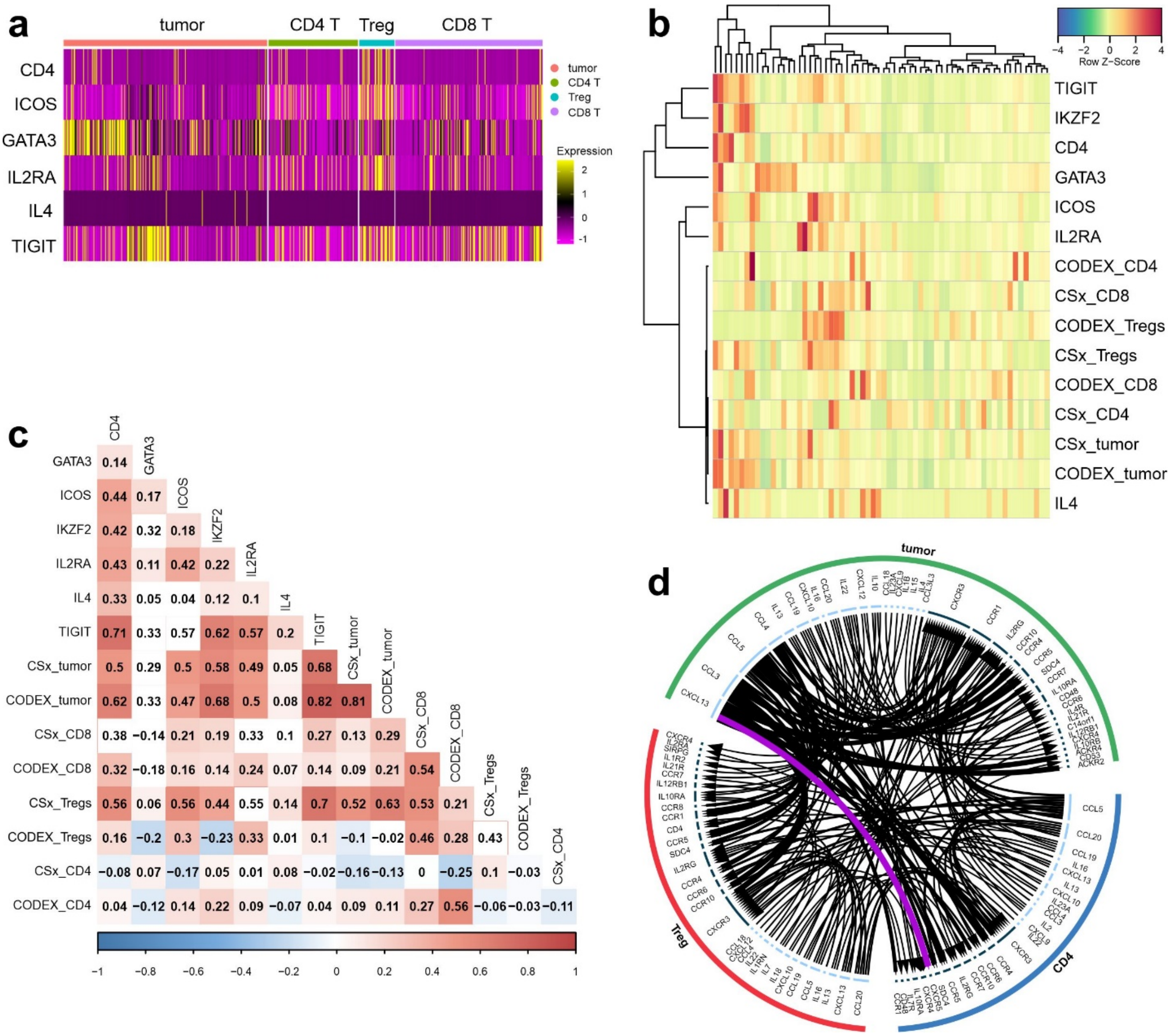

Supplementary Figure 8. CIBERSORTx deconvolves reactive tumor infiltrating lymphocytes (TILs) and tumor cells in bulk RNA-seq data. **a**, Heatmap of *CD4*, *ICOS*, *GATA3*, *IL2RA*, *IL4*, and *TIGIT* expression in CD4 T cells, CD8 T cells, Tregs, and tumor cells from the Gaydosik et al.[2] CTCL scRNA-seq dataset. Data were log normalized and row scaled for plotting. **b**, Heatmap of bulk log2 normalized gene expression (TPM) for *CD4*, *ICOS*, *GATA3*, *IL2RA*, *IL4*, and *TIGIT* as well as the frequency of reactive tumor infiltrating lymphocytes and tumor cells in bulk RNA-seq and CODEX for individual patients. **c**, Correlogram of bulk log2 normalized gene expression (TPM) and the cell-type frequencies. The correlation of CSx and CODEX detected cell-type frequencies demonstrates that CSx can accurately unmix bulk RNA-seq data with respect to reactive tumor infiltrating lymphocytes and tumor cells (see **Fig. 5h** for the correlation of other cell types). **d**, Circos plot of receptor-ligand interactions for chemokines and cytokines between CD4 T cells, tumor cells, and Tregs generated using the published CTCL scRNA-seq dataset[2]. The top 150 receptor-ligand interactions, based on the 50 most expressed receptor and ligand genes in the three cell-types analyzed, are depicted. A strong interaction was observed between *CXCL13* on tumor cells and *CXCR5* on $CD4^+$ T cells (purple line), but it was not statistically significant. Source data are provided as a Source Data file.

Supplementary Table 1a. Patient cohort.

Patient ID	Age at screening (years)	Diagnosis	Disease stage	Prior therapies	Response status	mSWAT change	Biopsy timepoints	C02 (weeks)	Response / progression time (weeks)	EOT time (weeks)	Death time (weeks)	Follow-up time (weeks)
1	76.14	MF	IIIA	3	Responder	-99.25	Pre, EOT			104		142
2	60.59	MF	IIIB	4	Responder	-89.31	Pre, EOT			104		151
3	52.01	SS	IVA	3	Non-responder	5.74	Pre, EOT			98	98	98
4	85.14	SS	IVA	4	Non-responder	-12	Pre, EOT			104	142	142
5	75.24	MF	IIB	6	Responder	-80.49	Pre, EOT			104		103
6	46.74	MF	IVA	9	Non-responder	-10.74	Pre, C02, EOT	3		25		25
7	46.71	MF	IIIB	3	Non-responder	134.85	Pre, EOT			9		9
8	67.44	SS	IIIB	1	Non-responder	198.46	Pre, EOT			104	121	121
9	77.65	SS	IVA	6	Responder	-90	Pre, C02, EOT	3		71		71
10	66.40	MF	IIIA	2	Responder	-74.21	Pre			N/A	119	119
11	72.23	SS	IVA	3	Responder	-100	Pre, Response		22	104		171
12	72.59	SS	IVA	5	Non-responder	-22.97	Pre, Progression		15	15		15
13	44.10	SS	IVA	1	Responder	-100	Pre, C02, Response, EOT	3	15	104		179
14	63.69	MF	IB	5	Non-responder	-2.76	Pre, C02, Response, EOT	3	10	92	92	92

MF: mycosis fungoides
SS: Sézary syndrome
mSWAT: modified Severity-Weighted Assessment Tool
Pre: biopsy obtained pre-treatment
C02: biopsy obtained prior to cycle 2 (3 weeks)
Response/Progression: biopsy obtained at point of response or progression
EOT: biopsy obtained at end of trial

Supplementary Table 1b. Tissue microarray layout.

TMA spot	Patient ID	Biopsy timepoint
1	1	Pre_1
2	1	Pre_2
3	1	EOT_1
4	1	EOT_2
5	2	Pre_1
6	2	Pre_2
7	2	EOT_1
8	2	EOT_2
9	3	Pre_1
10	3	Pre_2
11	3	EOT_1
12	3	EOT_2
13	4	Pre_1
14	4	Pre_2
15	4	EOT_1
16	4	EOT_2
17	5	Pre_1
18	5	Pre_2
19	5	EOT_1
20	5	EOT_2
21	6	Pre_1
22	6	Pre_2
23	6	EOT_1
24	6	EOT_2
25	7	Pre_1
26	7	Pre_2
27	7	EOT_1
28	7	EOT_2
29	8	Pre_1
30	8	Pre_2
31	8	Pre_3
32	8	EOT_1
33	8	EOT_2
34	8	EOT_3
35	9	Pre_1
36	9	Pre_2
37	9	EOT_1
38	9	EOT_2
39	10	Pre_1
40	10	Pre_2
41	11	Pre_1
42	11	Pre_2
43	11	Resp_1
44	11	Resp_2
45	12	Pre_1
46	12	Pre_2
47	12	Resp_1
48	12	Resp_2
49	6	C02_1
50	6	C02_2
51	13	Pre_1
52	13	Pre_2
53	13	Resp_1
54	13	Resp_2
55	13	EOT_1
56	13	EOT_2
57	14	Pre_1
58	14	Pre_2
59	14	Resp_1
60	14	Resp_2
61	14	Resp_3
62	14	EOT_1
63	14	EOT_2
64	14	EOT_3
65	14	C02_1
66	14	C02_2
67	9	C02_1
68	9	C02_2
69	13	C02_1
70	13	C02_2

Biopsy timepoint:
Pre: biopsy obtained pre-treatment
C02: biopsy obtained prior to cycle 2 (3 weeks**)**
Response/Progression: biopsy obtained at point of response or progression
EOT: biopsy obtained at end of trial

Supplementary Table 2. Targets.

Antibody target	Company	Catalog #	Clone	Oligonucleotide	Flourophore	Working dilution	Exposure time	Reaction cycle	Reaction channel
CD1a	Novus Biologicals	NBP2-34698	O10+CA1/711	43	Cy5	1:100	1/2s	20	4
CD2	Biolegend	300202	RPA-2.10	25	Cy5	1:25	1/2s	7	4
CD3	Cell Marque	custom	MRQ-39	77	Cy5	1:100	1/2s	17	4
CD4	Abcam	ab181724	EPR6855	20	ATTO550	1:100	1/2s	9	3
CD5	BD Biosciences	555350	UCHT2	75	ATTO550	1:50	1/2s	8	3
CD7	Cell Marque	custom	MRQ-56	63	ATTO550	1:100	1/2s	19	3
CD8	Cell Marque	custom	C8/144B	8	Cy5	1:50	1/5s	18	4
CD11b	Abcam	ab216445	EPR1344	28	Cy5	1:50	1/2s	13	4
CD11c	Abcam	ab216655	EP1347Y	49	ATTO550	1:50	1/2s	12	3
CD15	BD Biosciences	559045	MMA	14	Alexa488	1:200	1/10s	7	2
CD16	Cell Signaling Technology	custom	D1N9L	26	ATTO550	1:100	1/2s	13	3
CD20	Novus Biologicals	NBP2-54591	rIGEL/773	48	ATTO550	1:200	1/4s	11	3
CD25	Cell Marque	custom	4C9	24	ATTO550	1:100	1/2s	10	3
CD30	Cell Marque	custom	BerH2	57	ATTO550	1:25	1/2s	7	3
CD31	Novus Biologicals	NBP2-47785	C31.3+C31.7+C31.10	68	ATTO550	1:200	1/8.5s	25	3
CD34	Novus Biologicals	NBP2-34713	QBEnd/10	38	ATTO550	1:100	1/4s	23	3
CD38	Abcam	ab176886	EPR4106	66	ATTO550	1:100	1/2s	24	3
CD45	Novus Biologicals	NBP2-34528	B11+PD7/26	56	ATTO550	1:400	1/8.5s	20	3
CD45RA	BD Biosciences	555486	HI100	72	Cy5	1:50	1/2s	19	4
CD45RO	Biolegend	304202	UCH-L1	2	ATTO550	1:100	1/4s	22	3
CD56	Cell Marque	custom	MRQ-42	29	Cy5	1:50	1/2s	10	4
CD57	Biolegend	322325	HCD57	30	ATTO550	1:200	1/4s	21	3
CD68	Biolegend	916104	KP-1	70	Cy5	1:100	1/4s	23	4
CD69	R&D Systems	AF2359	polyclonal	36	ATTO550	1:200	1/2s	18	3
CD71	Cell Marque	custom	MRQ-48	3	Cy5	1:100	1/5s	22	4
CD138	Thermo Fisher Scientific	MA1-10091	B-A38	76	ATTO550	1:100	1/8.5s	26	3
CD162	Novus Biologicals	NBP2-80921	HECA-452	46	Cy5	1:200	1/8.5s	12	4
CD163	Novus Biologicals	NB110-40686	EDHu-1	45	Cy5	1:200	1/3s	26	4
CD164	BD Biosciences	551296	N6B6	69	Alexa488	1:200	1/2s	3	2
CD194	Biolegend	359402	L291H4	55	ATTO550	1:100	1/2s	14	3
Beta-catenin	BD Biosciences	610154	14	51	Cy5	1:50	1/2s	21	4
BCL-2	Cell Marque	custom	124	41	ATTO550	1:50	1/2s	17	3
Collagen IV	Abcam	ab6586	polyclonal	33	Cy5	1:200	1/4s	24	4
Cytokeratin	Biolegend	628602	C11	67	Alexa488	1:200	1/5s	6	2
DRAQ5	Cell Signaling Technology	custom	N/A	N/A	Cy5	1:100	1/8.5s	29	4
EGFR	Cell Signaling Technology	custom	D38B1	58	ATTO550	1:25	1/2s	15	3
FoxP3	Invitrogen	14-4777-80	236A/E7	61	ATTO550	1:100	1/4s	3	3
GATA3	Cell Marque	custom	L50-823	60	Cy5	1:100	1/2s	3	4
Granzyme B	Abcam	ab219803	EPR20129-217	81	Alexa488	1:200	1/8.5s	8	2
HLA-DR	Abcam	ab215985	EPR2692	65	ATTO550	1:200	1/4s	16	3
Hoechst 33342	Thermo Fisher Scientific	62249	N/A	N/A	DAPI	1:600	1/175s	all	1
ICOS	Cell Signaling Technology	custom	D1K2T	74	Cy5	1:100	1/2s	16	4
IDO-1	Cell Signaling Technology	custom	D5J4E	59	Cy5	1:25	1/2s	14	4
Ki-67	BD Biosciences	556003	B56	6	Cy5	1:100	1/5s	6	4
LAG-3	Cell Signaling Technology	custom	D2G4O	42	Cy5	1:25	1/2s	9	4
Mast cell tryptase	Abcam	ab2378	AA1	44	ATTO550	1:200	1/80s	27	3
MMP-9	Biolegend	819701	L51/82	62	Alexa488	1:400	1/3s	9	2
MMP-12	Abcam	ab137444	polyclonal	80	Cy5	1:100	1/2s	27	4
MUC-1	NSJ Bioreagents	V2372SAF	955	15	Alexa488	1:100	1/2s	4	2
p53	Cell Marque	custom	DO7	52	ATTO550	1:50	1/2s	4	3
PD-1	Cell Signaling Technology	custom	D4W2J	23	Cy5	1:50	1/2s	11	4
PD-L1	Cell Signaling Technology	custom	E1L3N	11	ATTO550	1:50	1/2s	6	3
Podoplanin	Biolegend	916606	D2-40	32	Cy5	1:200	1/3s	25	4
T-bet	Cell Signaling Technology	custom	D6N8B	5	ATTO550	1:100	1/2s	5	3
Vimentin	BD Biosciences	550513	RV202	7	Alexa488	1:200	1/4s	5	2
VISTA	Cell Signaling Technology	custom	D1L2G	79	Cy5	1:50	1/2s	15	4

Supplementary Table 3a. Interferon gamma gene score.

Gene	Coefficient
IFNG	-0.30685
HLA-DRA	-0.35484
IDO1	-0.36499
STAT1	-0.45212
CXCL10	-0.46207
CXCL9	-0.47832

Supplementary Table 3b. Transforming growth factor beta gene score.

Gene	Coefficient
IL6ST	0.48031
PDGFRB	0.40325
TGFBR2	0.39434
TNFRSF1A	0.39151
PDGFA	0.25311
KIT	0.25002
FLT4	0.24409
IFNGR1	0.10497
TNFRSF14	0.03628
ACVR1	-0.03375
TGFB1	-0.04924
TNFRSF10B	-0.15319
LIF	-0.17415
IL4R	-0.20508

Supplementary Table 3c. Immune activation gene score.

Gene	Coefficient
CXCL9	0.28056
CCL5	0.27507
GZMH	0.27229
EOMES	0.26916
GZMK	0.26379
CD27	0.25655
TNFRSF9	0.25375
IL2RG	0.24956
PRF1	0.24024
FASLG	0.23683
IL2RA	0.22805
IFNG	0.21895
ICOS	0.20233
CD40	0.18924
CD40LG	0.18919
IL17RA	0.14444
IL12B	0.13478
TNFSF9	0.12999
CD28	0.12862
CX3CR1	0.11662
TNFRSF4	0.11086
GZMB	0.07612
CCR7	0.06002
IL23A	0.05400

Supplementary Table 3d. Immunosuppression gene score.

Gene	Coefficient
TGFB1	0.31255
ENTPD1	0.30707
IL24	0.30658
CXCL1	0.30076
IL6	0.29989
TGFBR1	0.27375
IL10	0.24295
HAVCR2	0.20469
PDGFRA	0.20016
HLA-G	0.18822
TGFBRAP1	0.18282
PVT1	0.16990
PDGFRB	0.16723
LINC00473	0.16219
CXCL3	0.16065
TIGIT	0.15326
LIF	0.14962
CXCL12	0.12772
CD274	0.12391
TIAF1	0.11831
LAG3	0.10954
DNM3OS	0.02112
NIFK-AS1	-0.00826
IL4	-0.04139
GNAS-AS1	-0.05070
HOTAIR	-0.06027
PDCD1LG2	-0.08477
GAS5	-0.14413

Supplementary Table 3e. Cytotoxicity gene score.

Gene	Coefficient
NKG7	0.39045
PRF1	0.35018
TNF	0.34146
GZMH	0.32965
TBX21	0.31663
GZMK	0.31526
GZMM	0.29080
ID2	0.26046
IFNG	0.25047
GNLY	0.22788
GZMB	0.18149

Supplementary Table 3f. Tumor therapy resistance score.

Gene	Coefficient
IL2RB	0.30377
BATF	0.29598
IL21R	0.28415
CCND2	0.26500
RGS16	0.25940
MYO7A	0.25910
SLA	0.25436
ANP32E	0.23795
MTHFD2	0.21538
IL10	0.21012
TRIB2	0.20521
RARRES3	0.19264
GNLY	0.19238
DUSP5	0.18767
C1GALT1	0.15656
BCL2	0.15272
EHD1	0.13197
HSPD1	0.13011
DAD1	0.12384
MMP12	0.08265
IL26	0.07073
IL22	0.06240
P4HB	-0.05212
TCN1	-0.07340
SFTPD	-0.12905
TGFBR3	-0.18280

Supplementary Table 4a. Markers of CIBERSORTx cell-types.

Cell-types	Marker genes	CIBERSORTx cell-types
B cells & plasma cells	*MS4A1, LTB, CD79A, CD79B*	B and plasma cells
T cells	*CD3E, CD3D, CD274*	CD4 T, CD8 T, Tregs, tumor cells
Macrophages & dendritic cells	*AIF1*	Macrophages, dendritic cells
Keratinocytes	*KRT1, KRT14*	Epithelium
Vasculature	*CD43, PECAM1, CLDN5*	Vasculature
Fibroblasts	*COL1A1, SFRP2*	Stroma
Pericytes	*RGS5, ACTA2*	

Supplementary Table 4b. T cell and tumor cell markers for CIBERSORTx.

T cell markers (reactive CD4 T, CD8 T, Tregs)				Tumor markers			
CD4	*FOXP3*	*IL2*	*STAT6*	*ACTG1*	*CDK1*	*KIAA0101*	*PRDX1*
CD7	*GATA3*	*IL2RA*	*TBX21*	*ANP32B*	*CDK6*	*MCTS1*	*PSMB2*
CD8A	*GZMA*	*IL4*	*TGFB1*	*ATP5C1*	*CENPE*	*MKI67*	*RAN*
CD8B	*GZMB*	*ITGAE*	*TIGIT*	*BCL2*	*CENPM*	*MYC*	*RANBP1*
CD27	*HAVCR2*	*LAG3*	*TNF*	*BCL2L12*	*DUT*	*NPM1*	*SET*
CD44	*ICOS*	*PDCD1*	*TRAC*	*BIRC3*	*FOS*	*NUSAP1*	*SMC4*
CD69	*IFNG*	*PRF1*	*TRBC1*	*BIRC5*	*HMGN1*	*PCNA*	*STMN1*
CD160	*IKZF2*	*RORC*	*TRBC2*	*CCNA2*	*HMMR*	*PIM2*	*TOP2A*
CTLA4	*IL10*	*RUNX3*	*TRDC*	*CCND1*	*HN1*	*PLK1*	*TOX*
EOMES	*IL17A*	*STAT3*	*TRGC1*	*CDC20*	*IGF2*	*PPA1*	*TSC22*
FASLG	*IL1B*	*STAT4*	*TRGC2*	*CDCA8*	*IL2RA*	*PPIA*	

REFERENCES

1 Khodadoust, M. S. *et al.* Pembrolizumab in Relapsed and Refractory Mycosis Fungoides and Sezary Syndrome: A Multicenter Phase II Study. *J Clin Oncol* **38**, 20-28, doi:10.1200/JCO.19.01056 (2020).

2 Gaydosik, A. M. *et al.* Single-Cell Lymphocyte Heterogeneity in Advanced Cutaneous T-cell Lymphoma Skin Tumors. *Clin Cancer Res* **25**, 4443-4454, doi:10.1158/1078-0432.CCR-19-0148 (2019).
