# Supplementary material for: Immune cell topography predicts response to PD-1 blockade in cutaneous T cell lymphoma
Source: Nat Commun. 2021 Nov 18;12:6726. doi: 10.1038/s41467-021-26974-6 (PMC8602403; doi:10.1038/s41467-021-26974-6)
Supplement: Supplementary file 2 — Description of Additional Supplementary Files [file 41467_2021_26974_MOESM2_ESM.pdf]

Title: Supplementary Data 1.
Description: Marker expression, X/Y cell coordinates, cell-type annotation, and cellular neighborhood assignment for all segmented single-cells identified by CODEX.

Title: Supplementary Data 2.
Description: Raw counts of aligned transcripts for every gene in the transcriptome per tissue microarray spot.

Title: Supplementary Data 3.
Description: Tissue microarray key with corresponding RNAseq label.

Title: Supplementary Data 4.
Description: Minimal distances between cell-types in CODEX data.

Title: Supplementary Data 5.
Description: Minimal distances between cell-types in Vectra data.

Title: Supplementary Data 6.
Description: Gene expression of CIBERSORTx-resolved tumor cells per tissue microarray spot.

Title: Supplementary Data 7.
Description: Differentially expressed genes for the 10 annotated cluster.

Title: Supplementary Data 8.
Description: CIBERSORTx signature matrix.
