# Supplementary material for: Immune cell topography predicts response to PD-1 blockade in cutaneous T cell lymphoma
Source: Nat Commun. 2021 Nov 18;12:6726. doi: 10.1038/s41467-021-26974-6 (PMC8602403; doi:10.1038/s41467-021-26974-6)
Supplement: Supplementary file 11 — Reporting Summary [file 41467_2021_26974_MOESM11_ESM.pdf]

nature research

Corresponding author(s): Garry P. Nolan

Last updated by author(s): 9/17/2021

Reporting Summary

Nature Research wishes to improve the reproducibility of the work that we publish. This form provides structure for consistency and transparency in reporting. For further information on Nature Research policies, see our Editorial Policies and the Editorial Policy Checklist.

Statistics

For all statistical analyses, confirm that the following items are present in the figure legend, table legend, main text, or Methods section.

n/a	Confirmed	
☐	☒	The exact sample size (*n*) for each experimental group/condition, given as a discrete number and unit of measurement
☐	☒	A statement on whether measurements were taken from distinct samples or whether the same sample was measured repeatedly
☐	☒	The statistical test(s) used AND whether they are one- or two-sided<br>*Only common tests should be described solely by name; describe more complex techniques in the Methods section.*
☐	☒	A description of all covariates tested
☐	☒	A description of any assumptions or corrections, such as tests of normality and adjustment for multiple comparisons
☐	☒	A full description of the statistical parameters including central tendency (e.g. means) or other basic estimates (e.g. regression coefficient) AND variation (e.g. standard deviation) or associated estimates of uncertainty (e.g. confidence intervals)
☐	☒	For null hypothesis testing, the test statistic (e.g. *F*, *t*, *r*) with confidence intervals, effect sizes, degrees of freedom and *P* value noted<br>*Give P values as exact values whenever suitable.*
☒	☐	For Bayesian analysis, information on the choice of priors and Markov chain Monte Carlo settings
☐	☒	For hierarchical and complex designs, identification of the appropriate level for tests and full reporting of outcomes
☒	☐	Estimates of effect sizes (e.g. Cohen's *d*, Pearson's *r*), indicating how they were calculated

Our web collection on statistics for biologists contains articles on many of the points above.

Software and code

Policy information about availability of computer code

Data collection	CODEX imaging driver software (available from Akoya Biosciences, v1.29.0.1) and NextSeq system suite software (available from Ilumina, v2.20.0.422)

Data analysis	In house image processing scripts: https://github.com/NolanLab/CODEX<br>In house image clustering scripts: https://github.com/NolanLab/vortex<br>In house cellular neighborhood scripts: https://github.com/NolanLab/NeighborhoodCoordination<br>In house SpatialScore script: https://github.com/NolanLab/SpatialScore<br>CellEngine: https://cellengine.com<br>In house RNA-seq data processing scripts: https://github.com/jwfoley/3SEQtools<br>RNA-seq data processing scripts: https://github.com/alexdobin/STAR (STAR v2.7.3a)<br>CIBERSORTx: https://cibersortx.stanford.edu (CSx v1.0)<br>HALO software 2.0<br>LassoCV object in scikit-learn python package v0.22<br>R version 3.4.3, R packages:<br>lmerTest v3.1.2<br>lme4 v1.1.21<br>modelbased v0.1.2 (github.com/easystats/modelbased)<br>ggplot2 v3.3.0<br>ggrepel v.0.8.1<br>esayROC interface v1.3.1 (http://www.biosoft.hacettepe.edu.tr/easyROC/)<br>bootstrap-coupled estimation (DABEST) package v0.3.0<br>Seurat v3.1.4<br>GraphPad Prism v8

For manuscripts utilizing custom algorithms or software that are central to the research but not yet described in published literature, software must be made available to editors and reviewers. We strongly encourage code deposition in a community repository (e.g. GitHub). See the Nature Research guidelines for submitting code & software for further information.

Data

Policy information about availability of data

All manuscripts must include a data availability statement. This statement should provide the following information, where applicable:

- Accession codes, unique identifiers, or web links for publicly available datasets
- A list of figures that have associated raw data
- A description of any restrictions on data availability

CODEX imaging data generated in this study have been deposited in the ImmunoAtlas public repository (https://immunoatlas.org/NOLN/210920-1). RNA-seq data generated in this study have been deposited in the GEO database under accession code GSE162137 (https://www.ncbi.nlm.nih.gov/geo/query/acc.cgi?acc=GSE162137). Data tables can be downloaded from Mendeley (https://data.mendeley.com/datasets/3gmvy3bcmk/1). Source data are provided with this manuscript.

Field-specific reporting

Please select the one below that is the best fit for your research. If you are not sure, read the appropriate sections before making your selection.

☒ Life sciences ☐ Behavioural & social sciences ☐ Ecological, evolutionary & environmental sciences

For a reference copy of the document with all sections, see nature.com/documents/nr-reporting-summary-flat.pdf

Life sciences study design

All studies must disclose on these points even when the disclosure is negative.

Sample size	This was an exploratory study using existing FFPE tissue specimens from a previously published clinical trial (Khodadoust, M. S. et al. J Clin Oncol; 2020, doi:10.1200/JCO.19.01056). Given the observation nature of our study, we did not perform statistical analyses to predetermine sample size. The sample size of patients was limited by FFPE sample availability and the feasibility of assay throughput. Sample size was considered sufficient to represent the clinical trial population. Patient outcome measures (i.e., mSWAT change and survival analyses) showed expected effects between responders and non-responders.
Data exclusions	Patient samples that did not contain at least 1 cubic mm of FFPE tissue were excluded due to insufficient material; this criteria was pre-established. Samples with fewer than 10 cells per tissue microarray core were excluded from marker expression or cell-type frequency, owing to insufficient representation; this criteria was not pre-established. Sequencing libraries were excluded if <40% of their transcripts were within a 165-500 bp range, owing to likely primer dimers and poor quality data; these criteria were pre-established.
Replication	For CODEX, 3 experimental replicates were performed on consecutive tissue sections. As detailed in the manuscript, experiment #1 had the highest mean cell density per tissue microarray spot (Sup. Fig. 1d) and was therefore selected for further processing and analysis. The SpatialScore biomarker that was identified with CODEX was replicated and validated using the Vectra platform (1 experiment using a tissue microarray containing 70 tissue cores from 14 patients). Further replication studies were not conducted.
Randomization	Patient samples were allocated into responder and non-responder experimental groups based on their overall response rate to pembrolizumab therapy, as determined in the initial clinical trial publication using consensus global response criteria (Khodadoust, M. S. et al. J Clin Oncol; 2020, doi:10.1200/JCO.19.01056). Randomization is not relevant to the analysis of observational data.
Blinding	All data acquisition was conducted blinded to associated clinical and molecular data.

Reporting for specific materials, systems and methods

We require information from authors about some types of materials, experimental systems and methods used in many studies. Here, indicate whether each material, system or method listed is relevant to your study. If you are not sure if a list item applies to your research, read the appropriate section before selecting a response.

Materials & experimental systems

n/a	Involved in the study
☐	☒ Antibodies
☒	☐ Eukaryotic cell lines
☒	☐ Palaeontology and archaeology
☒	☐ Animals and other organisms
☐	☒ Human research participants
☐	☒ Clinical data
☒	☐ Dual use research of concern

Methods

n/a	Involved in the study
☒	☐ ChIP-seq
☒	☐ Flow cytometry
☒	☐ MRI-based neuroimaging

Antibodies

Antibodies used

Antibodies are detailed in Supplementary Table 2, which includes the following information:
Antibody target Company Catalog # Clone Oligonucleotide Flourophore Dilution Exposure time Reaction cycle Reaction channel
CD1a Novus Biologicals NBP2-34698 O10+CA1/711 43 Alexa647 1:100 1/2s 20 4
CD2 Biolegend 300202 RPA-2.10 25 Alexa647 1:25 1/2s 7 4
CD3 Cell Marque custom MRQ-39 77 Alexa647 1:100 1/2s 17 4
CD4 Abcam ab181724 EPR6855 20 ATTO550 1:100 1/2s 9 3
CD5 BD Biosciences 555350 UCHT2 75 ATTO550 1:50 1/2s 8 3
CD7 Cell Marque custom MRQ-56 63 ATTO550 1:100 1/2s 19 3
CD8 Cell Marque custom C8/144B 8 Alexa647 1:50 1/5s 18 4
CD11b Abcam ab216445 EPR1344 28 Alexa647 1:50 1/2s 13 4
CD11c Abcam ab216655 EP1347Y 49 ATTO550 1:50 1/2s 12 3
CD15 BD Biosciences 559045 MMA 14 Alexa488 1:200 1/10s 7 2
CD16 Cell Signaling Technology custom D1N9L 26 ATTO550 1:100 1/2s 13 3
CD20 Novus Biologicals NBP2-54591 rIGEL/773 48 ATTO550 1:200 1/4s 11 3
CD25 Cell Marque custom 4C9 24 ATTO550 1:100 1/2s 10 3
CD30 Cell Marque custom BerH2 57 ATTO550 1:25 1/2s 7 3
CD31 Novus Biologicals NBP2-47785 C31.3+C31.7+C31.10 68 ATTO550 1:200 1/8.5s 25 3
CD34 Novus Biologicals NBP2-34713 QBEnd/10 38 ATTO550 1:100 1/4s 23 3
CD38 Abcam ab176886 EPR4106 66 ATTO550 1:100 1/2s 24 3
CD45 Novus Biologicals NBP2-34528 B11+PD7/26 56 ATTO550 1:400 1/8.5s 20 3
CD45RA BD Biosciences 555486 HI100 72 Alexa647 1:50 1/2s 19 4
CD45RO Biolegend 304202 UCH-L1 2 ATTO550 1:100 1/4s 22 3
CD56 Cell Marque custom MRQ-42 29 Alexa647 1:50 1/2s 10 4
CD57 Biolegend 322325 HCD57 30 ATTO550 1:200 1/4s 21 3
CD68 Biolegend 916104 KP-1 70 Alexa647 1:100 1/4s 23 4
CD69 R&D Systems AF2359 polyclonal 36 ATTO550 1:200 1/2s 18 3
CD71 Cell Marque custom MRQ-48 3 Alexa647 1:100 1/5s 22 4
CD138 Thermo Fisher Scientific MA1-10091 B-A38 76 ATTO550 1:100 1/8.5s 26 3
CD162 Novus Biologicals NBP2-80921 HECA-452 46 Alexa647 1:200 1/8.5s 12 4
CD163 Novus Biologicals NB110-40686 EDHu-1 45 Alexa647 1:200 1/3s 26 4
CD164 BD Biosciences 551296 N6B6 69 Alexa488 1:200 1/2s 3 2
CD194 Biolegend 359402 L291H4 55 ATTO550 1:100 1/2s 14 3
Beta-catenin BD Biosciences 610154 14 51 Alexa647 1:50 1/2s 21 4
BCL-2 Cell Marque custom 124 41 ATTO550 1:50 1/2s 17 3
Collagen IV Abcam ab6586 polyclonal 33 Alexa647 1:200 1/4s 24 4
Cytokeratin Biolegend 628602 C11 67 Alexa488 1:200 1/5s 6 2
DRAQ5 Cell Signaling Technology custom N/A N/A Alexa647 1:100 1/8.5s 29 4
EGFR Cell Signaling Technology custom D38B1 58 ATTO550 1:25 1/2s 15 3
FoxP3 Invitrogen 14-4777-80 236A/E7 61 ATTO550 1:100 1/4s 3 3
GATA3 Cell Marque custom L50-823 60 Alexa647 1:100 1/2s 3 4
Granzyme B Abcam ab219803 EPR20129-217 81 Alexa488 1:200 1/8.5s 8 2
HLA-DR Abcam ab215985 EPR2692 65 ATTO550 1:200 1/4s 16 3
Hoechst 33342 Thermo Fisher Scientific 62249 N/A N/A DAPI 1:600 1/175s all 1
ICOS Cell Signaling Technology custom D1K2T 74 Alexa647 1:100 1/2s 16 4
IDO-1 Cell Signaling Technology custom D5J4E 59 Alexa647 1:25 1/2s 14 4
Ki-67 BD Biosciences 556003 B56 6 Alexa647 1:100 1/5s 6 4
LAG-3 Cell Signaling Technology custom D2G4O 42 Alexa647 1:25 1/2s 9 4
Mast cell tryptase Abcam ab2378 AA1 44 ATTO550 1:200 1/80s 27 3
MMP-9 Biolegend 819701 L51/82 62 Alexa488 1:400 1/3s 9 2
MMP-12 Abcam ab137444 polyclonal 80 Alexa647 1:100 1/2s 27 4
MUC-1 NSJ Bioreagents V2372SAF 955 15 Alexa488 1:100 1/2s 4 2

p53 Cell Marque custom DO7 52 ATTO550 1:50 1/2s 4 3
PD-1 Cell Signaling Technology custom D4W2J 23 Alexa647 1:50 1/2s 11 4
PD-L1 Cell Signaling Technology custom E1L3N 11 ATTO550 1:50 1/2s 6 3
Podoplanin Biolegend 916606 D2-40 32 Alexa647 1:200 1/3s 25 4
T-bet Cell Signaling Technology custom D6N8B 5 ATTO550 1:100 1/2s 5 3
Vimentin BD Biosciences 550513 RV202 7 Alexa488 1:200 1/4s 5 2
VISTA Cell Signaling Technology custom D1L2G 79 Alexa647 1:50 1/2s 15 4

As detailed in the Methods section, the following antibodies were used for Vectra mIHC and post Vectra CXCL13 staining:
Antibody target Company Catalog # Clone Host Concentration OPAL Flour Position
CD8 DAKO M7103 144B Mouse 0.8 ug/ml 520 1
CD25 Cell Marque 125M-14 4C9 Mouse 0.17 ug/ml 540 2
CD3 Thermo RM-9107 SP7 Rabbit 0.06 ug/ml 570 3
PD-1 Abcam ab137132 EPR4877(2) Rabbit 1 ug/ml 650 4
CD7 Cell Marque 107M-24 MRQ-56 Mouse 1.18 ug/ml 690 5
CD4 Epitomics AC0173A EP204 Rabbit 0.08 ug/ml 480 6
FoxP3 eBiosciences 14-4777-82 236A/E7 Mouse 5 ug/ml 620 7

Antibody target Company Catalog # Clone Host Concentration
CXCL13 R&D Biosciences AF801 polyclonal Goat 0.5 ug/ml
Goat ImmPress HRP Secondary Vector Labs MP-7405 n/a Horse Ready-to-use

Validation

Expected antibody staining patterns were confirmed in a variety of tissues, including cutaneous T cell lymphoma skin tissue. Details of vendor validation and use in previous publications are available at https://antibodyregistry.org, Schurch, C.M., et al. Cell; 2020, doi:10/1016/j.cell.2020.07.005, and Phillips, D., et al. Frontiers Immunology; 2021; doi: 10.3389/fimmu.2021.687673.

Human research participants

Policy information about studies involving human research participants

Population characteristics

This study used de-personalized FFPE skin specimens from a previously published CITN-10 phase II clinical trial (Khodadoust, M. S. et al. J Clin Oncol; 2020, doi:10.1200/JCO.19.01056) on adults with a clinicopathologically confirmed diagnosis of mycosis fungoides (MF) or Sézary syndrome (SS) (clinical stage IB to IV) that had relapsed, was refractory to, or had progressed after at least one standard systemic therapy and were treated with pembrolizumab therapy. All patients included in the clinical trial provided written informed consent for their tissues to be used for research purposes.

Patient demographics and covariants are detailed in Sup. Table 1a, with relevant details included below:
Patient ID Age at screening (years) Gender Diagnosis Disease stage Prior therapies Response status
1 76.14 Male MF IIIA 3 Responder
2 60.59 Male MF IIIB 4 Responder
3 52.01 Male SS IVA 3 Non-responder
4 85.14 Female SS IVA 4 Non-responder
5 75.24 Male MF IIB 6 Responder
6 46.74 Male MF IVA 9 Non-responder
7 46.71 Female MF IIIB 3 Non-responder
8 67.44 Male SS IIIB 1 Non-responder
9 77.65 Female SS IVA 6 Responder
10 66.40 Male MF IIIA 2 Responder
11 72.23 Male SS IVA 3 Responder
12 72.59 Male SS IVA 5 Non-responder
13 44.10 Female SS IVA 1 Responder
14 63.69 Male MF IB 5 Non-responder

Recruitment

Patient samples used in this study were selected as a retrospective cohort based on the availability of FFPE tissues from the previously published CITN-10 phase II clinical trial (Khodadoust, M. S. et al. J Clin Oncol; 2020, doi:10.1200/JCO.19.01056). Adults with a clinicopathologically confirmed diagnosis of mycosis fungoides (MF) or Sézary syndrome (SS) (clinical stage IB to IV) that had relapsed, was refractory to, or had progressed after at least one standard systemic therapy were recruited and consented for treatment with pembrolizumab and donation of skin tissue samples. Exclusion criteria for participation in the clinical trial included an age of less than 18 years, central nervous system disease, active autoimmune disease, previous exposure to any anti-PD-1, anti-PD-L1, or anti-PD-L2 therapy, or treatment with radiotherapy or other anti-cancer agents within 15 weeks of the pre-treatment biopsy. No biases relevant to the aims of this study were present as the patient characteristics were not found to differ between responder and non-responder patients, as shown in Sup. Fig. 1b.

Ethics oversight

The use of fully de-personalized FFPE patient tissues for this research study was approved by the Stanford University IRB Administrative Panels on Human Subjects in Medical Research (HSR 46894).

Note that full information on the approval of the study protocol must also be provided in the manuscript.

Clinical data

Policy information about clinical studies

All manuscripts should comply with the ICMJE guidelines for publication of clinical research and a completed CONSORT checklist must be included with all submissions.

Clinical trial registration	The current study involves retrospective analysis of tissue samples collected from previously published clinical trial NCT02243579 (Khodadoust, M. S. et al. J Clin Oncol; 2020, doi:10.1200/JCO.19.01056).
Study protocol	The full study protocol for this previously published clinical trial is available at https://clinicaltrials.gov and Khodadoust, M. S. et al. J Clin Oncol; 2020, doi:10.1200/JCO.19.01056.
Data collection	As part of the previously published clinical trial, tissue samples were collected at a tertiary hospital/clinic setting from 2014-2018 (Khodadoust, M. S. et al. J Clin Oncol; 2020, doi:10.1200/JCO.19.01056).
Outcomes	The primary end point for this previously published clinical trial was overall response rate by consensus global response criteria (Khodadoust, M. S. et al. J Clin Oncol; 2020, doi:10.1200/JCO.19.01056).
